# A Novel Social Network Approach to Measure Intersectional Stigma Among Latino Men Who Have Sex With Men in San Diego, California (NEXUS): Protocol for a Longitudinal Cohort Study

**DOI:** 10.2196/72334

**Published:** 2026-02-27

**Authors:** Laramie R Smith, Angel B Algarin, Eileen V Pitpitan, Heather A Pines, Nicole K Kelly, Carl Latkin, Aaron Gutierrez, Maryam Hussain, Francisco Soto, Ricardo Vazquez Jr, Stevie Juarez, Albert Genegaling, Rosalinda Rodriguez, Anthony Cirilo, Juan Esparza, Saul Cruz, Erick Jimenez, Ulises Reyes, Veronica Moore, Malek Guerbaoui, Beth Davenport, Jeannette Aldous, Katherine Penniga, Kenyatta Parker, Britt Skaathun

**Affiliations:** 1 Department of Medicine, University of California, 9500 Gilman Drive, #0507, San Diego, CA, 92093-0507, United States, 1 858 822 1462, 1 858 534 6084; 2 Center for Health Promotion and Disease Prevention, Arizona State University, Tempe, AZ, United States; 3 School of Social Work, San Diego State University, San Diego, CA, United States; 4 Herbert Wertheim School of Public Health and Human Longevity Science, University of California, San Diego, CA, United States; 5 School of Public Health, San Diego State University, San Diego, CA, United States; 6 Bloomberg School of Public Health, Johns Hopkins University, Baltimore, MD, United States; 7 San Ysidro Health, San Diego, CA, United States; 8 The San Diego LGBT Community Center, San Diego, CA, United States; 9 Rollins School of Public Health, Emory University, Atlanta, GA, United States

**Keywords:** intersectional stigma, social networks, latino MSM, HIV prevention, HIV stigma, PrEP use, HIV testing, men who have sex with men, pre-exposure prophylaxis

## Abstract

**Background:**

Latino men who have sex with men (LMSM) account for a disproportionate and growing number of HIV diagnoses in the United States. Intersectional stigma remains a key driver of HIV inequities; however, most quantitative intersectional stigma measures are limited and do not consider the larger social context.

**Objective:**

NEXUS is a longitudinal cohort study that will use social network methods and theory to rigorously measure intersectional stigma among LMSM and quantify the longitudinal association between intersectional stigma and HIV prevention outcomes.

**Methods:**

We will prospectively enroll 500 HIV-negative LMSM in San Diego, California, and follow participants over 1 year. At baseline and every 6 months thereafter (Month 0, Month 6, and Month 12), participants will complete an interviewer-administered social network inventory and a self-administered survey to collect information on their social networks (alter types, size, and characteristics) and HIV prevention engagement (HIV testing and pre-exposure prophylaxis use), respectively. Information on HIV prevention engagement will also be abstracted from medical records. Intersectional stigma will be operationalized as a multilevel latent variable comprised of observed measures of anticipated and enacted stigma experienced by a participant from an alter toward the participant’s Latino, masculine, and sexual identities. Multilevel structural equation modeling will be used to estimate the longitudinal association between intersectional stigma, HIV testing, and pre-exposure prophylaxis use, considering potential mediators and moderators.

**Results:**

NEXUS recruitment began in June 2021, and as of March 11, 2025, a total of 482 participants had been enrolled. Enrollment is planned to end by May 2025, with baseline results expected late 2025 and through the following year. Data collection for our prospective study aims is expected to be complete in June 2026, with data analysis and expected results published later that year.

**Conclusions:**

NEXUS will advance quantitative intersectional stigma measurement using a novel social network approach. This study will identify intervention targets to reduce HIV inequities among LMSM and mitigate the harms of intersectional stigma in this population.

## Introduction

Inequities in HIV acquisition among Latino men who have sex with men (LMSM) are increasing in the United States [1-3]. From 2018 to 2022, the number of HIV diagnoses among US LMSM rose dramatically (22% increase), despite declining or remaining stable among men who have sex with men of other ethno-racial groups (non-Hispanic White: 5% decrease, multiracial: 32% decrease, and non-Hispanic Black: no change) [4]; underscoring the need for targeted prevention efforts in this multiply marginalized group. The US HIV epidemic among LMSM is primarily localized in 4 states—California, Texas, Florida, and New York—with the largest number of new HIV diagnoses occurring in California [2]. San Diego County, California, in particular, has been named one of 50 priority jurisdictions by the US Ending the HIV Epidemic initiative [5]. Together, these jurisdictions account for over 50% of new HIV diagnoses in the country [5]. Despite biomedical advances in HIV prevention (eg, pre-exposure prophylaxis [PrEP]) [6], substantial barriers remain and reinforce existing inequities in prevention for LMSM, especially in high-incidence areas such as San Diego County [7,8]. Stigma is one such barrier that greatly undermines efforts to reduce HIV incidence through many different pathways [9], such as by impairing mental health and influencing substance use. While this is true of all populations, the effects of stigma on HIV are even more deleterious among multiply marginalized groups, such as LMSM [8,10-12].

Stigma is a social process that maintains health inequities, often experienced as anticipated (expecting unjust treatment), enacted (experiencing unjust acts perpetrated by others), and internalized (applying negative stereotypes, beliefs, and feelings toward oneself) stigma mechanisms [9,13,14]. However, stigma related to a marginalized component of one’s identity or characteristics rarely occurs in isolation. Instead, people often experience multiple, overlapping forms of stigma (ie, intersectional stigma) via stigmatizing interactions [15,16]. Applied to HIV prevention with LMSM, intersectional stigma reflects how stigma toward multiple stigmatized identities—ethnicity, masculinity, and sexuality—may interact with HIV stigma in diverse social contexts (eg, social relationship types and identity expression) [15-17]. For example, LMSM may contend with racism from non-Latino sexual partners and homophobia and machismo (ie, historical and cultural expectations of men taking pride in and performing hypermasculine gender roles) [18] from Latino family members, increasing fear of further rejection if one acquires HIV. LMSM also vary in the degree to which they present these identities to others (eg, acculturation, outness, and masculinity), influencing the degree to which they might experience intersectional stigma. Despite recognition that the social context of intersectional stigma matters, these contexts are rarely measured when evaluating stigma as a barrier to HIV prevention [19].

Despite the substantial focus that HIV stigma has received in the literature, when an intersectionality lens is applied, it is too often used to describe HIV stigma experiences within historically marginalized populations. This work typically does not quantitatively assess or contextualize the effects of intersecting stigmas on the health of these populations. It often relies on analytical approaches that are not grounded in intersectionality theory (eg, using interaction terms in regression models) [20-22]. Experts emphasize that intersectional stigma has been primarily studied via qualitative methods, which are important for illustrating the lived experience of intersectional identities, but point to the promise that quantitative methods have in intersectionality research [19,23]. While there is no single best practice to measure and analyze intersectional stigma, methods that can assess the magnitude and effects of intersectional stigma on health behaviors, such as HIV testing and PrEP use, are needed to advance the development of intersectional stigma-responsive interventions and improve HIV outcomes [19,23].

Social network methods offer a promising and innovative approach to measuring intersectional stigma within the larger social context and provide an opportunity to shift the focus of stigma from the individual level toward upstream social factors. In particular, social network methods measure social contexts by examining how individuals (ie, LMSM) interact with and are affected by the people in their social networks (ie, “alters,” such as family, friends, sexual partners, and providers) [24]. Within social networks, stigma toward multiple identities is produced and experienced in relation to others (social network intersectional stigma exposure) [13]. Social network intersectional stigma mechanisms are important intervention targets, since alters’ social response to intersectional identities can (1) stigmatize LMSM (ie, intersectional stigma sources) and (2) foster LMSM’s resilience, reducing the negative impact of stigma (eg, identity affirmation and social support) [25]. Social network methods can help us learn about the social contexts where stigma toward single or multiple stigmatized identities interacts to affect health behaviors by provoking a deleterious or protective response [25].

**Figure 1.**
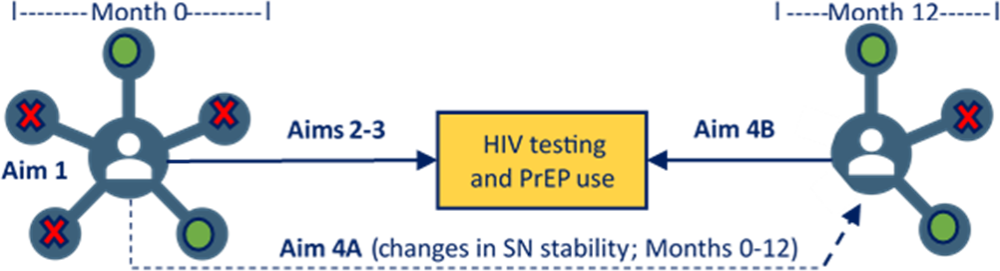
Visual representation of the NEXUS study aims 1‐4. Green color denotes affirming alter and red denotes stigmatizing alter. PrEP: pre-exposure prophylaxis; SN: social network.

Quantitatively measuring intersectional stigma is a well-documented challenge, and most methods either largely ignore the broader social context or fail to capture its nuances. However, stigma is fundamentally a social process, so incorporating social context is critical to understanding the complex interplay of these dynamics and to developing effective stigma-reduction interventions. Yet, to our knowledge, no studies have used a social network approach to measure intersectional stigma. We are therefore conducting a theory-informed, longitudinal cohort study of LMSM in San Diego County, California, called NEXUS—named to reflect the central point at which these social processes and contexts intersect. NEXUS aims to (1) identify how social network intersectional stigma manifests among LMSM by alter type (eg, family and sexual partner) and the degree to which LMSM express their marginalized identities (eg, acculturation, outness, and masculinity); (2) examine the longitudinal associations between social network intersectional stigma, HIV testing, and PrEP use; (3) explore network- and individual-level factors that may mediate (eg, social support and identity salience) and moderate (eg, network density and age) the association between social network intersectional stigma exposure on HIV testing and PrEP use; and (4) explore whether social network intersectional stigma exposure influences network stability (Aim 4A) and if changes in network stability are associated with increases in HIV testing and PrEP use (Aim 4B; Figure 1).

## Methods

### Study Design

**Figure 2.**
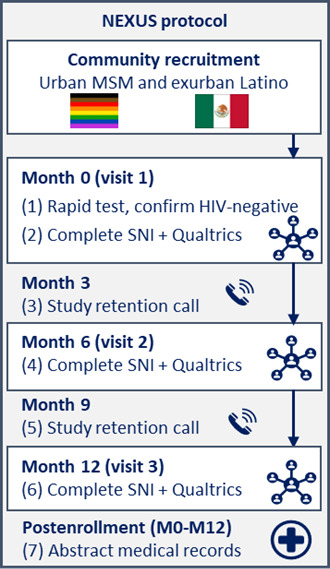
NEXUS study protocol. MSM: men who have sex with men; SNI: social network inventory.

NEXUS is a longitudinal, prospective cohort study that will aim to enroll 500 Latino gay, bisexual, and other men who have sex with other men (ie, LMSM) in San Diego, California, who are HIV negative (Figure 2). Our community-based recruitment will extend to urban lesbian, gay, bisexual, and transgender, and queer (LGBTQ) communities and exurban, predominantly Latino communities. Following a baseline rapid HIV test, HIV-negative participants will complete 3 study visits: baseline (Month 0), Month 6, and Month 12 postbaseline. A certified HIV tester and counselor will conduct baseline HIV tests as part of routine HIV prevention services offered in San Diego County. A trained bilingual (English-Spanish) and bicultural interviewer will lead study visits, and data will be collected electronically. At each study visit, we will collect self-report data captured through network- and individual-level survey methods (ie, a social network inventory [SNI] and Qualtrics survey [Qualtrics Software]) to study the longitudinal associations between intersectional stigma within egocentric social networks. HIV testing and PrEP use outcomes are obtained via self-report at each study visit and via medical records abstraction for the time between Month 0 and Month 12. As described in the “Data Management and Analysis” section below, data will be analyzed prospectively using multilevel structural equation modeling (MSEM). All study measures and protocols were first vetted by our HIV service community partners and bilingual LMSM who are also part of our study team and community advisory board (CAB), before piloting with 40 LMSM, to ensure they were acceptable and feasible to implement with the study cohort. Our prospective cohort study design will establish the degree to which LMSM are exposed to intersectional stigma within their social networks and how that exposure varies across different social relationships and contexts to affect HIV prevention behaviors over time.

### Study Population

**Figure 3.**
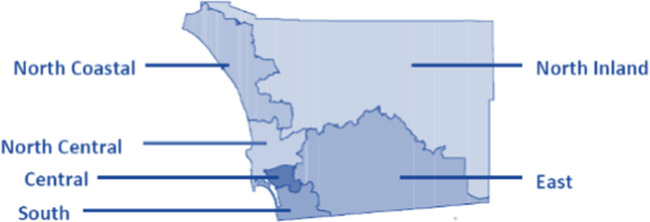
Map of San Diego County, California Health and Human Services Agency regions.

Eligible participants are LMSM in San Diego County, California, who meet the following criteria: (1) are at least 18 years of age, (2) identify as cisgender men, (3) identify as Latino or Hispanic, (4) are fluent in English or Spanish, (5) report sexual activity with men in the past 12 months, and (6) are HIV negative. We will aim to oversample LMSM aged 18‐44 years, among whom most incident HIV acquisitions are diagnosed [1]. The study will collaborate with 2 community partners who provide bilingual HIV services (the San Diego LGBT Community Center and a local HIV service provider) to enroll those living in geographically diverse regions of San Diego County (Figure 3). The San Diego LGBT Community Center is a large community-based organization located in Central San Diego County, a predominantly lesbian, gay, bisexual, transgender, and queer–identified urban area. The local HIV service provider is a large federally qualified health center with clinical sites primarily located in South and Southeast San Diego County. These sites are in proximity to the US-Mexico border and primarily serve patients in exurban Latino communities.

### An Integrated Theory-Informed Social Network Approach to Intersectional Stigma

#### Overview

In conceptualizing the NEXUS Study, we first articulated an integrated theory-informed social network approach to intersectional stigma, building on work by Pescosolido and Manago [26], who used contact theory [27] to articulate how stigma is likely experienced within social networks via 3 types of social processes (identity, influence, and affiliation). Social stigma [9,13,28], intersectionality [15,16,29], and social network [24,25] theories are inherently related, yet the linkages between them have not been well articulated. By articulating the integrated social stigma, social network, and intersectionality theories across these 3 social processes, we lay the foundation for NEXUS, a novel approach to assessing intersectional stigma as a socially networked phenomenon.

#### Identity Process

**Figure 4.**
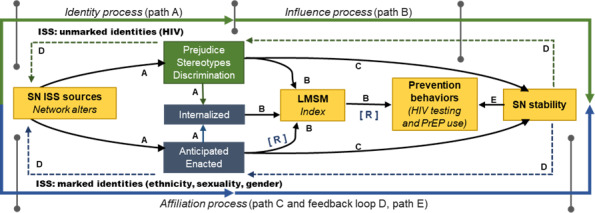
An integrated theory-informed social network approach measuring intersectional stigma among Latino men who have sex with men (LMSM) in the NEXUS study. ISS: intersectional stigma; PrEP: pre-exposure prophylaxis; SN: social network.

In brief, the identity process will specify from whom in the social network (intersectional stigma sources) anticipated and enacted stigma or affirmation can be experienced toward LMSM. The method will involve asking about each stigma (or affirmation) source (eg, family member, sexual partner) for each intersecting “marked” or socially devalued status (ie, ethnicity, masculinity, and sexuality; Figure 4, bottom half in blue) or “unmarked” stigmatized status they could acquire such as HIV (Figure 4, top half in green). Internalized stigma reflects the degree to which LMSM might turn the social devaluation process inward and self-stigmatize their ethnic, masculine, or sexual identities [9,26]. Stigma theory further specifies that the degree to which marked characteristics are visible (eg, skin tone and spoken language) or potentially concealable (eg, sexuality and gender expression) further influences how stigmatized statuses are experienced in social interactions [30-32]. NEXUS will focus on 3 specific intersectional identities (Latino, masculinity, and sexuality). These identities were selected because the scientific evidence linking ethnic-, gender-, and sexuality-related stigma to poorer HIV prevention outcomes among LMSM is strongest [33-38]. Additionally, this decision balances concerns of participant burden, as the addition of each identity exponentially increases the number of survey items, while selecting identities that are maximally inclusive of LMSM. However, an expanded intersectional identity focus is important for future programmatic research, once precedent for our novel social network approach is established.

#### Influence Process

The influence process will specify the association between anticipated and enacted intersectional stigma (via social interactions with network members) with LMSMs’ HIV prevention behaviors (ie, HIV testing and PrEP use) over time (Figure 4, path B) [26,30]. In the context of HIV research, an intersectionality lens is often applied to ensure co-occurring social statuses are observed at their distinct intersections and not in isolation from each other [15,39,40]; yet, approaches to this work vary [19]. Following guidance by Bowleg [41], many approach intersectionality as a blended identity (eg, stigma experienced as a gay-Latino-man). In this approach, the contributions of being Latino cannot be parsed out because it fundamentally shapes how life is experienced as a male sexual minority [42]. Yet, research on social identity centrality and salience suggests individuals may not experience these interlocking stigmatized identities equally in all social relationships or contexts [43].

#### Adaptive Process

Social network methods will enable NEXUS to model intersectional stigma exposure simultaneously across co-occurring social statuses to identify from whom and in what contexts intersectional stigma exposure affects future HIV prevention behaviors, while attending to both network-level factors (eg, social support from alters for HIV testing and network density) and individual-level factors (eg, identity salience and age) that may affect how (mediation) and for whom (moderation) exposure to social network intersectional stigma affects our outcomes of interest [26,44]. Relatedly, we articulate resilience as an adaptive (mediation) process [45] that may be experienced through interactions with network members who affirm and support traditionally stigmatized statuses or through an individual’s ability to rebound from and resist adversity. As such, we specify that the resilience process may facilitate LMSMs’ engagement in HIV testing and PrEP use despite high levels of social network exposure to intersectional stigma (Figure 4, path [R]) [46-48].

#### Affiliation Process

Relatedly, the affiliation process illustrates a dynamic social phenomenon through which we will articulate that social network exposure to intersectional stigma may affect the stability, density, and composition of the social network over time (Figure 4, path C) through the addition or removal of alters that affirm or reject LMSMs’ socially stigmatized statuses and thereby influence future social network exposure (Figure 4, feedback loop D) [26,44]. It will also reflect whether such changes in network stability are associated with increased HIV testing and PrEP use (Figure 4, path E) [26,49,50].

### Participant Procedures

#### Recruitment

We will use multiple bilingual nonrandom purposive sampling strategies to enroll the NEXUS cohort, including in-reach among clients and patients seeking services through our 2 community partners in San Diego County, California. Outreach strategies will include tabling at local community events and bars frequented by LMSM and using various social media platforms (eg, Instagram [Meta] and Facebook [Meta]) and dating apps (eg, Grindr [Grindr Inc] and Scruff [Perry Street Software]) with profiles tied to the NEXUS study and community partners.

#### Enrollment

Bilingual study staff will screen interested individuals to determine eligibility and, if needed, provide access to existing HIV testing services to confirm HIV seronegative status up to 14 days before the first study visit. Written informed consent, locator information to facilitate retention, and signed consent for a HIPAA (Health Insurance Portability and Accountability Act) authorization waiver and a medical release of information form will be collected at enrollment.

#### Study Visits

NEXUS participants will complete up to 3 study visits at Month 0 (baseline), Month 6, and Month 12, during which they will complete an interviewer-administered SNI via Network Canvas (Complex Data Collective) [51] and a self-administered individual-level survey via Qualtrics (Figure 2). While these visits were intended to be in person to allow for participants and interviewers to construct an interactive visual of the participants’ social network via Network Canvas on a large touch screen, NEXUS launched during the COVID-19 pandemic, necessitating the development of a virtual study visit protocol via Zoom (Zoom Communications, Inc). As physical distancing restrictions eased, participants had the option of in-person study visits. Baseline study visits typically last 2.5‐3 hours, while follow-up study visits last 1‐2 hours, depending on the participants’ social network size. Participants will be compensated as follows: Month 0: US $100, Month 3 (retention call; detailed below in the “Cohort Maintenance Strategies” subheading): US $10, Month 6: US $115, Month 9 (retention call): US $10, and Month 12: US $125.

### Data Collection Procedures

#### Overview

NEXUS will collect 3 primary types of data, detailed below in the “Social Network Inventory, Individual-level Survey, and Medical Records” sections, from cohort participants as outlined in Table 1. This includes an interviewer-administered SNI, a self-administered individual-level Qualtrics survey, and medical records data to document participants’ prospective HIV testing and PrEP use behaviors.

**Table 1.** NEXUS data collection sources and measures.

Source	Measure
Social network inventory	Social network name generators (alters) Network characteristics (density, size, and stability) Alter sociodemographics (age, gender, race, ethnicity, and sexuality) Alter relationship history (type, duration, frequency, and locations) Alter sexual history (as applicable) Identity awareness (ethnicity, masculinity, and sexuality) Anticipated intersectional stigma (ethnicity, masculinity, and sexuality) Avoidance of alters due to intersectional stigma (ethnicity, masculinity, and sexuality) Enacted intersectional stigma (ethnicity, masculinity, and sexuality) Other intersectional stigma exposure Intersectional affirmation and support from alters (ethnicity, masculinity, and sexuality) HIV testing and pre-exposure prophylaxis (PrEP) support COVID-19–related changes in social interactions
Individual-level Qualtrics survey	Sociodemographic information Latino identity (pride, centrality, salience, expression, and internalized stigma) Masculine identity (pride, centrality, salience, expression, and internalized stigma) Sexuality identity (pride, centrality, salience, expression, and internalized stigma) HIV prevention behaviors HIV and PrEP stigma Structural stigma Substance use behaviors Physical health Mental health Social desirability
Medical records	HIV testing PrEP visits

#### Piloting

In February 2021, before piloting data collection protocols and study measures, a community forum with simultaneous Spanish translation was held via Zoom, inviting potential participants and HIV prevention service providers to provide feedback on typical social network protocols to optimize the confidentiality of the network alters, while still allowing participants to identify and recall individual alters in the NEXUS SNI throughout each study visit and across follow-up study visits (described below in the “Social Network Inventory” section). From March to May 2021, we piloted our NEXUS study protocols, measures, and recruitment materials to iteratively optimize comprehension, reduce participant burden, minimize social desirability bias, and refine the linguistic and cultural relevance among 40 LMSM (21 English and 19 Spanish) using cognitive interviewing techniques where participants “think aloud” their interpretation of study materials and response process [52]. This allowed interviewers to document where revisions to study materials needed to be made. This one-time interview lasted up to 3 hours, and we alternated, starting with social network and individual-level measures to determine the best process for delivering the study protocol and eliciting network alters that are sources of anticipated and enacted intersectional stigma. From these procedures, we determined that the SNI should precede the individual-level survey. NEXUS pilot participants were remunerated US $100 for their participation.

#### Social Network Inventory

Trained bilingual interviewers will administer the SNI first using an interactive open-source software, Network Canvas [51]. Social networks will be enumerated using an egocentric network approach that ascertains information from participants on each member of their social network (ie, alters) [53]. We developed 7 name generators (ie, questions that probe participants to name people in their network) to identify alters that participants have interacted with in the past 6 months related to (1) social, (2) sexual, and (3) substance use interactions, as well as (4) emotional and (5) informational support that may increase exposure to HIV acquisition and HIV prevention, and (6 and 7) conflictual ties to capture interactions with alters who may not otherwise be named due to stigma (Table 2). We developed probes for each name generator to enhance the recall of alters. Interviewers were further trained to ask key questions if participants were having trouble naming anyone or listed fewer than 5 people for any given name generation, for example, asking, “Who else can I add?” and *“*What about people you interact with in a typical week?*”* Using the protocol developed in our community forum, which is typical of social network protocols and refined through the pilot phase, each alter will be identified by a first name or nickname that remains visible in Network Canvas for the rest of the interview, aiding recall of alters across study visits. To help distinguish between alters with similar first names and further support recall at follow-up visits, the first 4 letters of alters’ last names will be entered, but will not be visible unless that information needs to be accessed to aid in recall. Participants can also choose to add descriptors next to names that will be noted in parentheses (eg, coworker 1). If a participant does not know or declines to provide an alter’s first name or first 4 letters of the last name, NEXUS interviewers will be trained to enter “(9999)” and to have a brief discussion about how to refer to this alter in conversations and to input this information in the interview’s notes section programmed into Network Canvas. Information on the NEXUS alter generation protocol, including additional probes and Spanish translations, is available in Multimedia Appendix 1.

**Table 2.** Alter domains and name generator prompts used in the social network inventory among Latino men who have sex with men in the NEXUS study.

No	Alter domain	Name generator prompt
1	Social	“With whom did you hang out with or socialize regularly in person or through technology?”
2	Sexual	“With whom did you have oral, anal, or vaginal sex that was 18 years old or older?”
3	Substance use	“With whom did you drink alcohol with or use drugs with more than once?”
4	Emotional support	“With whom did you talk to about the MOST private and personal things in your life?”
5	Informational support	“From whom did you get advice about your health, HIV, or PrEP^a^?”
6	Conflictual tie: rejection	“Who makes comments (even as a joke) that make you think or feel like they don’t accept you for who you are?”
7	Conflictual tie: avoidance	“Who might you want to avoid because they say or do things that make you feel uncomfortable or unwelcome?”

a PrEP: pre-exposure prophylaxis.

Participants will then complete a sociogram to identify which alters know each other to characterize network density (ie, connectivity) [53]. If participants name more than 5 alters for a domain during the name generator process, we will ask them to identify the top 5 people who are most important to them for each domain. For the top 5 alters for each name generator, we will elicit key social network characteristics, including: sociodemographics (age, gender, race, and ethnicity), sexual orientation, relationship type (family, friend, sexual partner, health care provider, and other), relationship strength (very close, somewhat close, no longer close, and never close), proximity (resides in the San Diego-Tijuana border region), and perceived or known HIV and PrEP status of alter (Table 1). We will then assess the characteristics of social interactions with each alter by eliciting the structure (typical activities), frequency, and locations or venues of typical social interactions. Finally, we will assess the context of social interactions with alters related to anticipated and enacted intersectional stigma related to their ethnicity, masculinity, and sexuality, as well as other social reactions related to these socially stigmatized statuses (eg, alters affirming LMSM’s socially stigmatized statuses as an important part of who they are and LMSM avoiding alters because of their stigmatizing behavior), and social support for HIV testing and PrEP behaviors.

At follow-up visits, we will assess social network stability over time by collecting information for each alter named at baseline who was not named at subsequent visits, and each new alter added at follow-up visits to identify reasons for (1) not naming previous alters at follow-up and (2) not interacting with these alters at follow-up. For the top 5 alters named for each domain at follow-up, we will again assess social network characteristics, as well as social interaction characteristics and context as described above. Refer to Multimedia Appendix 2 for items assessing social network stability. Network Canvas is not designed, at this time, to support longitudinal social network data collection by retaining alters across interview protocols. As such, we developed a NEXUS longitudinal social network data integration protocol with multiple verification checks to generate alter reports from previous surveys to be input into Network Canvas by the study team ahead of subsequent study visits. In brief, this protocol is completed in three steps: (1) use SNI data from the previous interview to generate an individual alter report for each participant (ie, using Month 0 SNI data or Month 6 SNI data), (2) enter data from the alter report ahead of the participants’ upcoming scheduled interview (ie, Month 6 interview or Month 12 interview), and (3) at each follow-up visit with participants, assess what alters left or entered the network over time. Detailed instructions and best practices learned through the development and implementation of the NEXUS longitudinal social network data integration protocol, including sample syntax for running the alter reports, are provided in Multimedia Appendix 2 and can be easily adapted by other researchers using Network Canvas in longitudinal study designs to ensure data integrity while tracking alters across multiple study visits.

#### Individual-Level Survey

The individual-level survey will be conducted through Qualtrics and will collect a range of information, including sociodemographic factors, identity measures (Latino, masculinity, and sexuality), health behaviors, internalized stigma, and substance use. A full list of survey domains is provided in Table 1 and described in detail in the “Measures” subheading below.

#### Medical Records

Signed HIPAA authorization waiver and release of medical information forms specifically indicate participants agree to and request the release of HIV testing and PrEP-related information as part of their study participation. NEXUS participants will be able to enroll in the study if they decline to sign these forms, but, based on experience, we anticipate that few (<2% of participants) will opt out. These forms will facilitate access to HIV testing and PrEP visit data abstracted from participants’ medical records for 12 months following the NEXUS baseline visit via secure fax or encrypted email per the medical providers’ protocols. If no HIV testing or PrEP data are available or the participants’ records cannot be located by the provider, the provider will send documentation indicating these results, and we will record this response as “no medical records data available.” To offset the risks of missing medical records data, HIV testing and PrEP data will also be collected via self-report at each study interaction (Figure 2: Months 0, 3, 6, 9, and 12). Details on each of these outcomes are provided in the “Measures” subheading, but in brief, information on HIV testing date, HIV testing results, PrEP prescription coverage (dates of injection and dose provided or dates of pills prescribed, number of refills for pills, and number of pills per refill) and PrEP clinical care (visit dates with a PrEP-prescribing provider, laboratory tests performed, and laboratory test results) will be abstracted. The medical records data will be immediately deidentified, abstracted into a secure database, and linked to the participants’ unique study ID before analysis.

#### Cohort Maintenance Strategies

To facilitate retention, study staff will contact participants by phone at Month 3 and Month 9, three months after their first and second study visit, respectively (Figure 2). Retention calls will update participant locator information and assess whether HIV testing and PrEP use occurred and where those services were accessed since the last study visit.

### Measures

#### Overview

All measures will be captured at Month 0 (baseline), Month 6, and Month 12 study visits unless otherwise specified (Table 3). All measures will be available in both English and Spanish. When a validated Spanish version of a measure is not available, the measure will be translated from English to Spanish by a bilingual, bicultural Spanish speaker and back-translated by a second bilingual, bicultural member of the study team who is native to Mexico. Discrepancies will be discussed and resolved via consensus with a third bilingual Spanish speaker. The Spanish translation will then be reviewed by multiple bilingual individuals from both community partners, and refinements will be made through a working group meeting. Items related to stigmatized identities have been refined as needed to be culturally responsive to LMSM via feedback and discussions with LMSM from our study CAB, community partners, and the NEXUS study team. When needed, adaptations were made to ensure clarity or cultural responsiveness. Any adaptations made to these measures are noted below under the “Measures” subheading. Unless otherwise specified, measures will be available upon reasonable request from the corresponding author. Once measures are validated in the NEXUS sample, the English and Spanish versions will be published in the corresponding paper.

**Table 3.** Analytical measures and data sources by the NEXUS study’s primary aims.

Measure type and name		Source	Aims
Exposures			
Intersectional stigma		Social network inventory	1‐4
Alter type		Social network inventory	1
Identity expression: L^a^, M^a^, and S^a^		Qualtrics survey	1
Outcomes			
HIV testing		Medical records and Qualtrics	2‐4
PrEP^b^ use		Medical records and Qualtrics	2‐4
Social network stability		Social network inventory	4
Mediators^c^			
Resilience		Qualtrics survey	3
Medical mistrust		Qualtrics survey	3
Identity pride: L, M, and S		Qualtrics survey	3
Identity centrality: L, M, and S		Qualtrics survey	3
Identity salience: L, M, and S		Qualtrics survey	3
Internalized stigma: L, M, and S		Qualtrics survey	3
HIV stigma		Qualtrics survey	3
PrEP stigma		Qualtrics survey	3
Moderators^c^			
Network size		Social network inventory	3
Network density		Social network inventory	3
Network composition		Social network inventory	3
Nativity		Qualtrics survey	3
Local community affiliation		Qualtrics survey	3
Age group		Qualtrics survey	3
Covariates^d^			
Age		Qualtrics survey	1‐4
Race		Qualtrics survey	1‐4
Education		Qualtrics survey	1‐4
Income		Qualtrics survey	1‐4
Years living in San Diego County		Qualtrics survey	1‐4
Alcohol use		Qualtrics survey	1‐4
Substance use		Qualtrics survey	1‐4
Depression		Qualtrics survey	1‐4
Anxiety		Qualtrics survey	1‐4
HIV knowledge		Qualtrics survey	1‐4
PrEP willingness		Qualtrics survey	1‐4
HIV testing frequency		Qualtrics survey	1‐4
PrEP use history		Qualtrics survey	1‐4

a L: Latino, M: masculinity, and S: sexuality.

b PrEP: pre-exposure prophylaxis.

c Mediators and moderators as hypothesized.

d Covariates for each aim are measured at the individual level and will be identified from a directed acyclic graph, theory, or the extant literature.

#### Primary Exposures

##### Intersectional Stigma

Social network intersectional stigma will be operationalized as a multilevel latent variable comprising 6 observed scores that are specific to anticipated and enacted stigma mechanisms experienced from an alter toward a participant’s Latino, masculine, and sexual identities. Each observed score will reflect the mean response (at baseline) or individual response (at follow-up) given for each alter in response to the 6 mechanism-specific stigma exposures. By modeling observed scores from each alter, we can maximize variance in exposure to stigma across all alters, rather than truncating this variance into a composite score averaged across all alter types (eg, a mean score referencing all “family and friends”), as is common in traditional survey research. This approach allows us to capture how the magnitude, significance, and direction of association for these 6 mechanism-specific stigma exposures vary when simultaneously modeled in relation to different stigma sources (ie, family, friends, sexual partners, providers, and other alters), as well as participants’ self-reported identity expression (ie, the degree to which LMSM report their interests, attitudes, behaviors, and appearances as being typically Latino, masculine, or gay). Social network intersectional stigma will be measured using the SNI data. The NEXUS social network intersectional stigma items, response options, and Spanish translations are provided in Multimedia Appendix 3.

At baseline, anticipated intersectional stigma exposure will be assessed by asking, “In the future, how likely it is that each alter will ‘stereotype you,’ ‘judge you or look down on you’ (prejudice), and ‘treat you poorly or avoid you’ (discrimination) for each intersectional stigma identity” (3 items each and 9 items total). Responses will be recorded on a 5-point Likert-type scale (1=very unlikely, 2=unlikely, 3=it’s possible, 4=likely, and 5=very unlikely). Baseline enacted intersectional stigma exposure will be assessed by asking, “In the past, how often has each alter ‘stereotyped you’ ‘judged you or looked down on you’ (prejudice), and ‘treated you poorly or avoided you’ (discrimination) for each identity” (3 items each and 9 items total). Responses will be recorded on a 5-point Likert-type scale (1=never, 2=not often, 3=somewhat often, 4=often, and 5=very often).

For Month 6 and Month 12, we refined the measures to ask a single anticipated stigma item for each stigmatized identity (3 items total) and a single enacted stigma item for each stigmatized identity (3 items total). The single item asks how likely, in the future, each alter (anticipated), and how often, in the past, has each alter (enacted) “stereotyped you, judged you, treated you poorly, or avoided you” for each marginalized identity. No changes were made to the response options. While this change will limit our ability to compare the same social network intersectional stigma items across time, it was deemed necessary to reduce the SNI response burden following the baseline assessment and ensure sufficient retention at study follow-up. Specifically, each SNI item is asked for each alter; a difference of 18 total items per alter at baseline vs 6 total items per alter at follow-up can dramatically affect response burden, especially for larger networks. A direct comparison of social network intersectional stigma items across assessment points would be further limited by changes in social network composition over time, as individual alters may leave or enter the network at various time points. Notably, this change does not affect the primary study aims, which focus on the prospective associations between baseline social network exposure to intersectional stigma among LMSM and their future HIV testing and PrEP use. However, this change will allow us to compare the precision with which social network intersectional stigma exposure can be measured with multiple items per intersectional stigma mechanism compared with a more general single item per intersectional stigma mechanism in cross-sectional analyses. Future results can better inform under which circumstances greater measurement precision (and participant response burden) is recommended, enabling future studies to make an informed decision when selecting the multi-item or single-item social network intersectional stigma measurement approach that can be compared across time.

##### Stigma Sources

We will examine alter type to assess how social network intersectional stigma varies across diverse social relationships. Alter type will be modeled as a continuous observed variable for the proportion of alters identified as a specific alter type (eg, the number of family members out of the total network size) in the SNI. Alter characteristics will be assessed in the SNI. Alters will be categorized as (1) family, (2) friends, (3) sexual partners, or (4) other. Family members will be further categorized as parent, sibling, aunt, uncle, cousin, grandparent, nonblood family (including chosen family and family by marriage), or other. Sexual partners will be further categorized as someone in a serious relationship, someone you dated, a casual sexual partner (including “fuckbuddy” and “friends with benefits”), a one-night stand, or anonymous. Alters, first described as “other,” will be further categorized as a medical provider, therapist, neighbor, coworker, religious group member, or other.

##### Identity Expression

We will collect information on identity expression to assess how social network intersectional stigma varies in relation to LMSM’s expression of their intersectional identities. Identity expression will be modeled as continuous variables (mean scores) derived from Qualtrics responses related to the degree to which LMSM report their (1) self-view, (2) ideal-self, (3) interests, (4) attitudes, (5) behaviors, and (6) appearance as being typically Latino (ethnicity), masculine, and gay (sexuality). These items were adapted for ethnicity and sexuality from the 6-item Traditional Masculinity-Femininity scale [54]. A seventh item was added from Rider et al [55]: “Other people would describe me as….” to capture how LMSM perceive how others typically view them. Responses will be scored on a single bipolar 7-point Likert scale, ranging from 1 (very Latino) to 7 (not at all Latino), 1 (very masculine) to 7 (very feminine), and 1 (very gay) to 7 (very straight), respectively.

We added an exploratory measure of Latino identity expression using a single item from the Short Acculturation Scale for Hispanic people language subscale, “In which language(s) do you usually think?” with response options including 1 (only Spanish), 2 (more Spanish than English), 3 (both equally), 4 (more English than Spanish), and 5 (only English) [56], as a proxy for acculturation (ethnicity). We will measure expression or endorsement of hypermasculinity (masculine identity) using the 10-item machismo subscale from the existing Machismo and Caballarismo Scale [57]. Minor edits to language were made to make the questions more applicable to a nonheteronormative context (eg, replacing “wife” with “partner”). Finally, we will measure sexual identity expression as a single-item indicator of sexual orientation outness [58] on a 5-point Likert scale (range: 1=not at all open or out to 5=open or out to almost all people I know). The item was modified to reflect same-sex behaviors vs same-sex identity or attraction: *“*How open (out) are you as someone who is a man who has sex with other men?”

### Primary Outcomes

The primary outcomes will be HIV testing and PrEP use at Month 6 and Month 12. These outcomes will be ascertained from both the medical records (primary outcome) and self-reports during the Qualtrics survey (exploratory outcome). We will define HIV testing as an HIV test date occurring in the past 12 months. We will define PrEP use (1) as the proportion of the past 12 months during which daily oral or injectable PrEP was prescribed, and (2) as a binary variable indicating whether any PrEP was prescribed in the past 12 months, to better account for participants who may have opted for on-demand oral PrEP dosing. Our decision not to assess on-demand PrEP oral dosing (ie, 2-1-1) was based on the fact that this dosing schedule was not approved by the Centers for Disease Control and Prevention or the US Food and Drug Administration, though this decision may potentially lead to an undercount of PrEP use. However, we will be able to conduct a sensitivity analysis with participants who are prescribed any oral PrEP during the assessment period and who self-report that they are “currently taking PrEP” without specifying their dosing schedule at Months 0, 3, 6, 9, and 12. We will also assess social network stability as an outcome (exploratory), which will be modeled as a latent variable using the proportion of alters named at Month 0 in the SNI that were identified as either an intersectional stigma source or identity-affirming source, but either (1) not named at Month 6 or 12, or (2) named as a new alter at Month 6 or 12.

### Mediators

#### Identity-Related Measures

In line with Quinn and Earnshaw’s [59] construction of the stigmatized identity model*,* we will assess how LMSM construct the individual-level meaning of their ethnic, masculine, and sexual identities in terms of their magnitude (centrality and salience) and valence (pride and internalized stigma) in Qualtrics. Ethnicity, masculinity, and sexuality-related identity centrality will be measured using the 6-item Identity Centrality Scale [60]. Items will be articulated to specify participants’ “Latino or Hispanic,” “masculine,” and “sexual” identities, respectively. Responses will be provided on a 7-point Likert-type scale (1=strongly disagree to 7=strongly agree). We will measure identity salience (for Latino, masculine, and sexual identities) using Quinn and Chaudoir’s [61] single-item measure, and responses will be provided on a 7-point Likert-type scale (1=almost never, 2=several times a year, 3=once a month, 4=once a week, 5=a few times a week, 6=once a day, and 7=many times a day). Ethnicity-, sexuality-, and masculinity-related pride will be used as a measure of identity affirmation (positive valence). A single-item continuous measure will ask participants, “On a scale of 0-100, how much pride do you take in your (Latino, masculine, or sexual) identity?” The definition of ethnic pride by Upadhyayula et al [62] was used to inform the structure of these items.

#### Internalized Stigma

Internalized Latino-, masculinity-, and sexuality-related stigma will be used as a measure of identity self-rejection or devaluation (negative valence) and will be collected during the Qualtrics survey. For Latino-related stigma, we adapted the Devaluation of Own Group subscale from the Appropriated Racial Oppression Scale [63] to be more appropriate for Latino or Hispanic participants (8 items). After consulting with community partners, we created 3 additional questions that aimed to reflect emotional responses to one’s Latino or Hispanic ethnicity that were deemed to have a lower potential for social desirability bias than the scale’s original items. Responses will be given on a 7-point Likert-type scale (1=strongly disagree to 7=strongly agree).

After failing to find an internalized masculinity stigma scale that did not conflate sexuality and gender, we created a new masculinity-related internalized stigma measure for this study. Item content was informed through a 2-step process: first, we used the 5-item structure of the Revised Internalized Homophobia Scale (IHP-R) by Herek et al [64], but changed “sexuality” to “masculinity” or “femininity.” Next, we reworded items to mirror the framing of items from existing validated internalized stigma subscales [65]. Responses will be given on a 5-point Likert-type scale (1=strongly disagree to 5=strongly agree).

For sexuality-related stigma, we also adapted the IHP-R [64]. After consulting with community members, 2 items were revised using language from the 4-item internalized sexuality stigma measure of Earnshaw et al [66]. We made several other language revisions to ensure that questions better reflected the changes in social conventions against conversion therapy and to better reflect the language our participants might use. An additional sixth item was added based on community partner concerns that the “get help” to change one’s sexuality item might perform poorly due to lack of relevance, and that “feeling guilty” about one’s sexuality would be more relevant in our population. Responses will be given on a 5-point Likert-type scale (1=strongly disagree to 5=strongly agree).

#### Resilience

Potential mediators may also reflect social responses that may influence how social network intersectional stigma affects HIV testing and PrEP use (modeled as latent variables) in Qualtrics. We measure resilience using the 10-item Connor-Davidson Resilience Scale (CD-RISC 10) [67] to reflect an individual-level response to intersectional stigma on our outcomes of interest. However, an error occurred during survey creation: respondents were asked to indicate “how much you agree or disagree with the following statements about how you generally respond to challenging situations,*”* and response options were recorded as strength of agreement (1=strongly disagree to 5=strongly agree) instead of frequency (1=not true at all to 5=nearly all of the time). We will note this error and consider its implications in all future analyses using the CD-RISC 10 measure.

#### Medical Mistrust

Medical mistrust reflects another means of social response to structural manifestations of intersectional stigma that may influence our outcomes of interest. Medical mistrust toward health care systems (hospitals) will be measured in the Qualtrics survey at Month 0 and Month 6 using the 5-item Medical Mistrust Scale from LaVeist et al [68]. In line with this measure, 5 additional items were created in an attempt to capture a more intersectional LMSM experience related to ethnicity, language, masculinity, sexuality, and its disclosure. All questions will be asked on a 4-point scale (1=strongly disagree to 4=strongly agree).

#### Sexual Behaviors and HIV Prevention Support

We will assess sexual behaviors in the SNI as a relational process mediating the association between intersectional stigma and HIV testing and PrEP use (primary outcomes). For each sexual partner, we will assess contextual factors (eg, relationship type and how they met) and behaviors that may affect HIV-related transmission risk (eg, frequency of drinking or using drugs before sex, past 6-month anal and vaginal sexual partners, and anal or vaginal sex without a condom). HIV prevention norms will be assessed for all alters in terms of expressing favorable or unfavorable opinions for HIV testing and PrEP use, perceived social support from alters for HIV testing and PrEP use (informational, tangible, and emotional support), and factors that may affect the perceived credibility of the HIV prevention norms (alters’ perceived HIV, ART, and PrEP status).

#### HIV and PrEP Stigma

We will assess stigma toward HIV and PrEP in Qualtrics at Month 0 and Month 12 as a sociocognitive process mediating the relationship between intersectional stigma with HIV testing and PrEP use. We will measure PrEP stigma using Siegler’s 12-item PrEP Stigma Scale [69] and HIV stigma using the 13-item HIV Stigma Mechanisms Scale for people without HIV [70]. We adapted the HIV stigma scale in response to feedback from community partners and CAB members. Based on this feedback, we eliminated the need for reverse-coded items in the prejudice subscale and replaced 3 of the 4 discrimination subscale items that community members felt were outdated or potentially stigmatizing. Three new discrimination items were developed with our community partners to better reflect contemporary contexts in which LMSM may express HIV-related bias toward people with HIV (eg, dating apps, in-person dates, and HIV status disclosure in the dating context). One item in the stereotypes subscale was adapted to be more relevant to the LMSM context. Responses to both stigma scales will be given on a 5-point Likert-type scale (1=strongly disagree to 5=strongly agree).

### Moderators

Potential moderators will include social network and LMSM characteristics, such as network size (number of alters), network density (connections between alters), and network composition (alter characteristics; all measured in the SNI). Those measured in Qualtrics will include nativity (born in the United States), years lived in the United States, local community affiliation (urban men who have sex with men [MSM] community or exurban Latino community based on zip code), and age group (18‐24, 25‐34, 35‐44, and ≥45 years).

### Covariates

Covariates will be collected during the baseline Qualtrics survey and will include sociodemographic characteristics, such as age, race, skin tone [71], self-described sexual orientation, education, income, and years living in San Diego County. We will measure alcohol use with the Alcohol Use Disorders Identification Test-Concise (AUDIT-C) [72] and substance use with the World Health Organization’s Alcohol, Smoking and Substance Involvement Screening Test (ASSIST; version 3.0) [73]. Intimate partner violence will be assessed at baseline via 3 items adapted for this study [74-76]. Information on mental health (collected at Month 0 and Month 6) will include measuring symptoms of depression (using the 10-item Center for Epidemiologic Studies Depression [CESD-10] scale [77]), anxiety (using the Generalized Anxiety Disorder-7 Scale [GAD-7] [78]), and post-traumatic stress disorder using the Primary Care-Post-Traumatic Stress Disorder scale [79]. Social desirability will be assessed via RAND’s 5-item social desirability response set [80].

We adapted the SHIPP HIV Knowledge Scale [69] based on piloting feedback to measure HIV knowledge at Month 0, removing 3 items with limited variability reflecting (1) knowledge that people with HIV can look healthy, and misinformation that (2) there is an HIV vaccine, and (3) that a shower after sex reduces HIV risk. A new item was developed with community partners to measure knowledge of Undetectable=Untransmittable. PrEP knowledge will be assessed at Month 0 using a 13-item subscale from Walsh’s [81] PrEP Information-Motivation-Behavioral Skills model measure. Items were modified to add the word “pills” after the word PrEP to reduce conflating responses between oral vs injectable PrEP. Perceived HIV risk will be measured on a scale from 0 to 100 (0=no chance of getting HIV to 100=definitely). Other potential covariates may include HIV testing frequency, HIV testing barriers [82], PrEP barriers [83], and engagement in the PrEP care cascade [84,85]. Items related to COVID-19 (testing, vaccine status, vaccine willingness, and COVID-19–related fear [86]) will be assessed at Month 0.

### Data Management and Analysis

#### Data Structure and Software

This study will include multilevel data, where level 1 data are variables related to alters in a participant’s social network (eg, alter characteristics and intersectional stigma exposure from an alter). Alters will be nested within participants (level 2 data). Level 2 variables are related to LMSM (eg, participant characteristics) and aggregate network characteristics. SPSS (IBM SPSS Statistics) will be used to run descriptive statistics and univariate analyses to examine frequencies and distributions of the exposures, outcomes, mediators, moderators, and exploratory variables of interest. Appropriate nonparametric alternatives will be considered if parametric assumptions fail. Significance tests will be 2-sided (*P*≤.05). For adapted or newly created measures (outlined above under the “Measures” subheading), we will test validity and internal reliability before including measures in analyses for the study’s primary aims.

All study aims (1-4) will be analyzed in Mplus (Muthén and Muthén; version 8.1) using MSEM, a robust tool for modeling egocentric social network data, accounting for the dependence between level 1 data (eg, intersectional stigma exposure from alters) nested within level 2 data (eg, LMSM HIV testing and PrEP use) [87-89]. We will evaluate model fit using standard fit indices for all MSEMs (root-mean-square error of approximation ≤0.05, comparative fit index >0.95, and Tucker-Lewis index >0.95) [90]. Models may be adjusted to balance model fit, parsimony, and theory. Potential confounders for each aim will be identified from a directed acyclic graph [91] or identified via theory and the extant literature. If theoretically meaningful or statistically significant differences are observed across participants by recruitment site or geographic regions in San Diego County, these factors will be included as covariates in all final models.

#### Aim 1: Intersectional Stigma Measurement and Experiences

**Figure 5.**
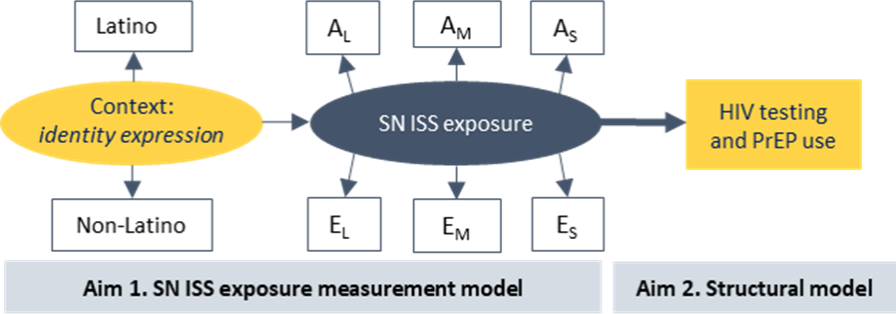
Hypothesized multilevel structural equation model for study aims 1 and 2. A: anticipated stigma; E: enacted stigma; SN: social network; social network; ISS: intersectional stigma; PrEP: pre-exposure prophylaxis; L: Latino identity; M: masculine identity; S: sexuality identity.

In the first aim, we will identify how social network intersectional stigma manifests at baseline (Month 0) by alter type and the degree to which LMSM express their intersectional identities (Figure 1). First, intraclass correlation coefficients (ICCs) will be examined for all level 1 variables to confirm sufficient variance for MSEM (ICC≥0.05). If the ICC is low (<0.05), indicating that the data are independent, level 1 data will be aggregated and treated as level 2 data (eg, group-mean centered) [92,93]. We will then construct a multilevel latent variable measurement model for social network intersectional stigma, composed of all observed scores (level 1) for anticipated and enacted stigma experienced for each intersectional stigma identity (Latino ethnicity, masculinity, and sexuality) at baseline (Figure 5).

After ensuring measurement model fit, we will fit separate structural models to examine the associations between the social network intersectional stigma latent variable and eight different level 2 contextual variables by alter type (family, friend, sexual partner, provider, other) and the degree to which LMSM express each identity (ethnicity, masculinity, and sexuality). For each a priori theory-informed structural model by partner type and by identity expression, we will control for Type I errors via a modified Bonferroni procedure for MSEM [94]. We will use the previously specified fit statistics, trim nonsignificant paths, and adjust the model (if necessary) using modification indices that align with a priori hypotheses.

#### Aim 2: Relationship Between Intersectional Stigma, HIV Testing, and PrEP Use

In aim 2, we will examine the longitudinal association between the social network intersectional stigma latent variable at baseline (Month 0) and HIV testing and PrEP use at the final study visit (Month 12; Figure 1). A separate structural model for each primary outcome of interest (HIV testing [binary] and PrEP use [continuous]; level 2) will be assessed for each of the 8 models tested in aim 1 (16 a priori models total) to examine which contexts (5 intersectional stigma sources and 3 intersectional identity expressions) best predict future HIV testing and PrEP use (Figure 5). This will be determined by model fit comparison (as specified above under the Data Structure and Software subheading), and the magnitude, direction, and significance of the outcome regressed on the social network intersectional stigma latent variable.

#### Aim 3: Mediation and Moderation

In aim 3, we will explore factors that may mediate and moderate the association between social network intersectional stigma with HIV testing and PrEP use (Figure 1). We will use Preacher’s [93] framework for assessing MSEM mediation models on level 2 outcomes (HIV testing and PrEP use). For each structural model developed in aim 2, we will fit our mediators of interest as a random effect separately for each social network (latent variables) and LMSM (observed score) response to stigmatized identities, HIV testing, and PrEP use. We will estimate the direct, indirect (level 2), and total effects for the proposed mediated processes. If there are multiple statistically significant mediators for an a priori structural model, each mediator will be entered into a single MSEM multiple mediation model to assess their independent effects.

For each structural model developed in aim 2, we will fit our moderators of interest by separately specifying an interaction term between social network intersectional stigma and each moderator. For models that demonstrate statistically significant moderation, we will conduct post hoc analyses to explore the level (by quartiles for continuous variables) at which the social network characteristic moderates the observed relationship between social network intersectional stigma, HIV testing, and PrEP use.

#### Aim 4: Social Network Stability

In aim 4, we will explore whether social network intersectional stigma exposure at baseline (Month 0) predicts changes in network stability across all study visits (Months 0‐12) and whether this change in network stability is associated with increased HIV testing and PrEP use at the final study visit (Month 12; Figure 1). A structural model will be assessed for each of the 8 social network intersectional stigma measurement models that test the path between social network intersectional stigma exposure at Month 0 on LMSMs’ social network stability latent variable at Month 12. To control for these associations, a separate (noncausal) path will be specified in which the social network stability variable at Month 12 is regressed on a latent variable comprised of observed scores for HIV testing and PrEP use at Month 12. Model fit, the magnitude, direction, and significance of the model paths will be examined.

#### Missing Data

Since NEXUS preferred sites comprise the majority of HIV service providers in Central and South San Diego County, we expect that the amount of missing medical records data for our primary outcomes will be minimal (<10%). Substantial efforts will be made to ensure Health Insurance Portability and Accountability Act authorization waivers and medical records release of information documents are updated and that participants complete follow-up. We will investigate the content (eg, HIV test dates and PrEP visits) and the type of missing data observed (eg, missing completely at random). However, a distinct advantage of using MSEM is that parameters are estimated with maximum likelihood (ML) estimation (for normal distributions) or ML with robust SEs, which is ML with robust SEs for nonnormal or ordinal data. ML is a rigorous method to handle missing data that yields unbiased parameter estimates, is appropriate for handling data missing completely at random or missing at random, and requires fewer assumptions about the data than multiple imputation [95]. In sensitivity analyses, we will (1) explore the impact of data missing not at random (eg, declining consent to obtain medical records), and (2) assess whether using HIV testing and PrEP use self-report data at Month 6 and 12 to supplement cases with null medical records may bias our findings.

#### Statistical Power

The following power calculations were conducted for aim 2, as aims 3 and 4 are exploratory, and aim 1 is a measurement model. Our target sample size of 500 LMSM will provide adequate statistical power (2-sided α=.05, 1-β≥.81) to detect the effect of social network intersectional stigma on HIV testing and PrEP use. As most intersectional stigma research with MSM is qualitative, we used published effect size estimates for race-related [33,34,96], sexuality-related [33,34,96,97], and gender-related stigma [98,99] among ethno-racially diverse MSM on HIV testing and sexual risk (σ=0.30-0.60). We were unable to locate estimates on PrEP use. We modeled a range for the ICC (0.50-0.90), assuming a high degree of shared norms toward intersectional stigma identities is observed by alter type (eg, sexual vs family alters) clustered within LMSM. A sample size of 500 will yield sufficient power for effect sizes greater than 0.40, or an effect size of 0.30 with a moderate (0.50) ICC. For observed ICC values <0.50, we may be underpowered to detect an effect. In response, we will model our 2-sided α (α=.05) for the number of participants enrolled (eg, N=500), along with our observed ICC, and assess our power to detect the observed effect to aid in the interpretation of study results.

#### Data Management

The program manager will review data monthly to track study enrollment and monitor data quality, allowing for timely adjustments if needed. To ensure high-quality data and fidelity to SNI administration, the first 10 baseline SNIs per study interviewer and a random sample of 5% of all SNI surveys thereafter will be audio-recorded for fidelity monitoring (ie, elicitation of alters from the index and completeness of SNI delivery).

### Ethical Considerations

The NEXUS study protocol was approved by the University of California, San Diego’s Institutional Review Board (protocol no 200712). Study protocols were solicited and modified in response to feedback from our 2 community partners and our study-specific CAB across all study phases. Our CAB comprises 4 bilingual self-identified LMSM who are diverse in terms of length of residence in San Diego County and knowledge of local HIV services. CAB members received a biannual honorarium of US $250 for their time and expertise. Per protocol, written informed consent was obtained from all enrolled NEXUS participants in their preferred language (English or Spanish), and all data will be de-identified to maximize participants’ privacy and confidentiality. Enrolled participants could earn up to US $360 for completing all NEXUS study activities.

## Results

The NEXUS study received funding in July 2020, with an expected end date of April 2025. Protocols were adapted to meet the changing COVID-19 response landscape. From March to May 2021, we piloted our NEXUS study protocols, measures, and recruitment materials. Cohort recruitment began in June 2021, and our anticipated enrollment rate was impacted by COVID-19. As of March 11, 2025, a total of 482 of 500 (96%) LMSM had enrolled in NEXUS and completed the baseline visit, 345 of 412 (84%) had completed the Month 6 visit, and 317 of 351 (90%) had completed the Month 12 visit. Enrollment is expected to end by May 2025, and data analysis for Aim 1 and other baseline characteristics is expected to begin in June 2025. Data collection for our prospective study aims is expected to be completed in June 2026, with data analysis and publication of expected results later that year.

## Discussion

### Anticipated Principal Findings

We anticipate that the NEXUS study will advance our understanding of how and from whom experiences of intersectional stigma vary in relation to LMSM social networks to affect HIV prevention outcomes. For example, from aim 1, we expect that we may observe the strongest associations with enacted masculinity and sexuality stigma when looking at network members who are family, but stronger associations with anticipated Latino and masculinity stigma when looking at network members who are sexual partners. Subsequently, aim 2 should inform us from whom these exposures to intersectional stigma are most strongly associated with PrEP use at Month 12, hypothesizing that we may observe stronger negative associations between PrEP use and intersectional stigma exposure from family than sexual partners. Aim 3 results should provide greater insights into what mediation processes may strengthen (ie, internalized masculinity stigma) or attenuate (ie, resilience) these negative associations with PrEP use, and for whom those associations are strongest (ie, LMSM with smaller social networks and LMSM who are younger). In contrast, aim 4 results should provide insights into the role network stability plays in HIV prevention, for example, greater stability among friend networks who are welcoming of LMSM may be associated with greater HIV testing at Month 12, compared to less stability among sexual partners who are stigmatizing.

These findings will be highly relevant to the US HIV epidemic, as LMSM experience a disproportionate risk of HIV acquisition, and intersectional stigma is widely recognized as a key barrier to eliminating this inequity [14,21,100,101]. Current approaches to quantitative intersectional stigma measurement are limited, and experts continually underscore that this remains a key challenge in the field [19,21]. Most existing measures of stigma toward intersectional identities are rarely measured in relation to one another or in relation to the social contexts that affect how and from whom intersectional stigma is experienced (ie, alter types and expression of stigmatized identities) [19,21,23]. The NEXUS study will use a social network approach to studying intersectional stigma. This methodology is highly innovative because it reflects a substantial departure from how intersectional stigma has been studied and potentially opens new opportunities to end the HIV epidemic. Specifically, NEXUS will allow us to identify the social contexts that affect how intersectional stigma is experienced by LMSM to affect HIV testing and PrEP use. It will also help elucidate the mechanisms that mediate or moderate the association between intersectional stigma exposure, HIV testing, and PrEP use. We will use results from NEXUS to develop novel intersectional stigma intervention targets for future piloting to reduce LMSM’s HIV incidence. In doing so, we also aim to promote resilience and reduce the harmful effects of intersectional stigma. Without understanding the larger social context of intersectional stigma and using this information to guide future work, HIV inequities will persist, and we will struggle to meet the national goals to end the HIV epidemic by 2030 [5].

### Limitations

However, this approach is not without its limitations, including the added response burden associated with a social network assessment of intersectional stigma. We hope that the ability to analyze and compare the cross-sectional associations between our multi-item mechanism-specific approach assessed at baseline (Month 0) and the more general single-item mechanism-specific approach assessed at follow-up (Months 6 and 12) can inform when or if this response burden can be attenuated while still capitalizing on the rich nature of social network data. Similarly, the COVID-19 pandemic coincided with the implementation of the NEXUS study, which required innovation in our intended recruitment and retention strategies and the development of more time-intensive virtual protocols. These strategies may have affected the types of LMSM we were able to reach with fewer in-person recruitment opportunities earlier on, but may have also increased the range of LMSM who agreed to participate, given the potential for greater perceived privacy and flexibility of our virtual protocols. These shifts may have further impacted study retention; however, our retention protocols and twice-monthly study team meetings with real-time retention monitoring and implementation of solutions-oriented retention strategies have allowed us to maintain an 84% and 90% retention rate at Month 6 and Month 12 follow-up visits based on the available data. Finally, medical mistrust may play a role in who agreed to enroll in the study and which enrolled participants consented to have their HIV testing and PrEP use data abstracted from their medical records and deidentified for analysis. Medical mistrust, along with variation in how medical records data are recorded across providers, is a limitation to be expected in any HIV-related study, especially those among ethno-racially marginalized groups. As such, the findings should be as generalizable as those of other published studies, and limitations in generalizability to the broader LMSM community will be noted in subsequent publications to guide our interpretation of the study results. Relatedly, we will assess if enrolled participants who declined to have their medical records abstracted, withdrew from the study for any reason, or were lost to follow-up varied in relation to their baseline medical mistrust scores, and if observed associations with HIV testing and PrEP use outcomes were moderated by medical mistrust at baseline to help guide our interpretation of study results.

### Conclusions

As NEXUS study results emerge, all findings will be interpreted in light of the extant literature, theory, and lived experiences of NEXUS study community partners, interviewers, and CAB members. In addition to traditional dissemination models (ie, peer-reviewed publications), we aim to disseminate study results through bilingual, in-person, and virtual forums with our community partners, hosted by our NEXUS interviewers, who worked hard to build and maintain trust and rapport with NEXUS participants. Additionally, we aim to develop brief infographics that summarize NEXUS study findings and their implications for supporting LMSM’s health and well-being, which can be disseminated via channels used for study recruitment, including our study website and local social media channels, tabling at local health fairs and bar-based HIV testing, and more broadly through our established relationships with scientific and community organizations in California and across the United States (eg, the California Center for HIV Syndemic Policy Research, the San Diego Center for AIDS Research, National Minority AIDS Council, the National Hispanic Science Network, and the National Latinx Conference on HIV, hepatitis C virus, and substance use disorder).

## Supplementary material

10.2196/72334 Multimedia Appendix 1 NEXUS social network name generator protocol.

10.2196/72334 Multimedia Appendix 2 NEXUS longitudinal social network data integration protocol.

10.2196/72334 Multimedia Appendix 3 NEXUS social network intersectional stigma–related items.

## Abbreviations

ASSIST: Alcohol, Smoking, and Substance Involvement Screening Test
AUDIT-C: Alcohol Use Disorders Identification Test-Concise
CAB: community advisory board
CD-RISC 10: 10-item Connor-Davidson Resilience Scale
CESD-10: 10-item Center for Epidemiologic Studies Depression
GAD-7: Generalized Anxiety Disorder-7 Scale
HIPAA: Health Insurance Portability and Accountability Act
ICC: intraclass correlation coefficient
IHP-R: Revised Internalized Homophobia Scale
LGBTQ: lesbian, gay, bisexual, and transgender, and queer
LMSM: Latino men who have sex with men
ML: maximum likelihood
MSEM: multilevel structural equation modeling
MSM: men who have sex with men
PrEP: pre-exposure prophylaxis
SNI: social network inventory
