# Supplementary material for: A Novel Social Network Approach to Measure Intersectional Stigma Among Latino Men Who Have Sex With Men in San Diego, California (NEXUS): Protocol for a Longitudinal Cohort Study
Source: JMIR Res Protoc. 2026 Feb 27;15:e72334. doi: 10.2196/72334 (PMC12954697; doi:10.2196/72334)
Supplement: Multimedia Appendix 1 [file resprot-v15-e72334-s001.docx]

NEXUS Name Generator Guide

BASELINE

**Q: Why are Name Generators SO important?**

**A: Because they tell us WHO is in people’s social networks and WHAT their relationships are like!**


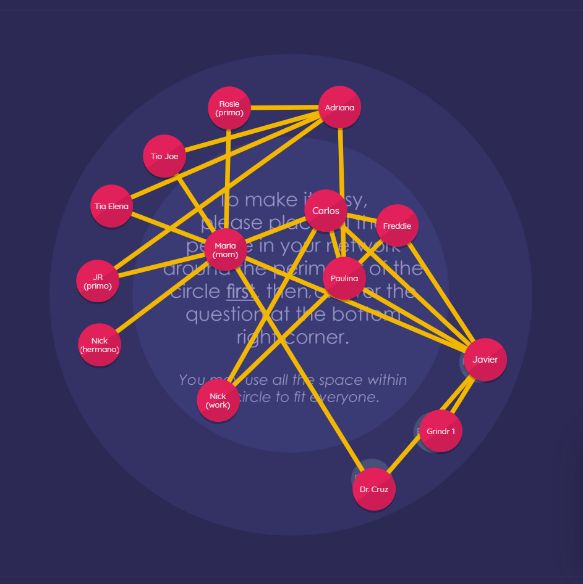

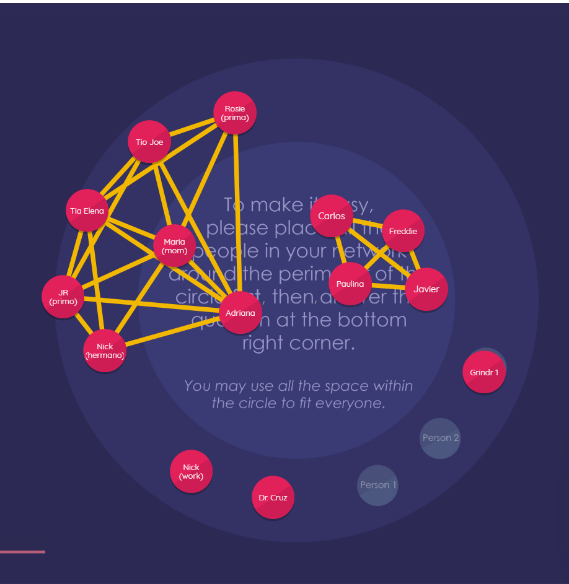


For example, in the figure above, these two networks have the same number of alters – but the one on the left shows someone that keeps family (top left) separate from their close group of friends (center right) with some alters such as co-workers, doctors, and some sex partners unconnected to everyone else. In contrast, the figure on the right shows family is a bit less interconnected with each other, but also some family, work colleagues, close friends, and sex partners are more interconnected. The experiences of the participants from these two social networks are likely VERY different, and we would never know that unless we probe for network members with equal enthusiasm across all name generator questions.

To make sure we can accurately describe participants’ social networks, we need to take our time with the name generator question, using the following tips:

- **If a participant lists fewer than 5 people or is having trouble naming anyone for a name generator question, try asking:**
  - *Who else can I add?*
  - *Please think back to the last time you [-- insert description --] was that with someone you have not listed yet?*
  - *What about the other people you have named already, does this apply to any of them?*
  - *What about people you interact with in-person in a typical week?*
  - *How about people you interact with on the phone, by text, social media or apps?*
- **NEXUS asks 7 name generator questions to illustrate predicants’ social networks over the past 6 months.**

Probes are listed below to help you think about WHAT KINDS of social relationships might be useful to explore.

These include:

- - People who provide social and informational support
  - People they socialize with
  - People who they have sex with and engage in substance use with
  - And **conflictual ties** – or people who they have strained or tenuous relationships with – these questions specifically try to assess potential sources of stigma
- **NOTE for Conflictual Ties (name generator 6 and 7):**

**Interviewers note:** probe for people who don’t accept them or who they specifically avoid because of their ethnic, masculine, or sexual identities.

- - For example, we don’t want people they avoid just because they might owe them money or people who don’t accept them because they voted Republican.

However, If they say**, ‘I don’t have anyone like this, I just cut those negative people out of my life,’ state, ‘**That’s OK, let’s name who those people are here because it seems, they had a negative impact!’

# SNI 1: NAME GENERATORS (Months 0)

**READ:** In this survey, we will talk about adults who are 18 years or older who you interacted with in the **past 6 months**. These can be adults you interacted with in-person, by phone or text, or through social media or dating apps. This includes adults who are family, friends, sexual or romantic partners. It can also include other adults like co-workers, neighbors, healthcare providers or people from your religious group.

This survey asks how you experience being a Latino man who has sex with other men in social interactions. Your answers will help us learn how these experiences might affect health promotion efforts like HIV prevention. Having names and demographic information of the people you interacted with will help us see how people in this study are connected to one another. For example, through mutual friends. It will also help us remember who we talked about today, the next time we meet. Please remember that everything you tell me is confidential and that we will protect the privacy of the people you mention. We will never at any time use this information to contact people.

It is OK to name people you have interacted with in the past 6-months for more than one of these next few questions.

En esta encuesta, hablaremos sobre adultos mayores de 18 años con los que interactuó en los **últimos 6 meses**. Estos pueden ser adultos con los que interactuó en persona, por teléfono o mensaje de texto, o a través de las redes sociales o aplicaciones de citas. Esto incluye a los adultos que son familiares, amigos, parejas sexuales o románticas. También puede incluir a otros adultos como compañeros de trabajo, vecinos, proveedores de atención médica o personas de su grupo religioso.

Esta encuesta pregunta cómo experimentas ser un hombre latino que tiene relaciones sexuales con otros hombres en las interacciones sociales. Sus respuestas nos ayudarán a saber cómo estas experiencias pueden afectar los esfuerzos de promoción de la salud como la prevención del VIH. Tener los nombres y la información demográfica de las personas con las que interactuó nos ayudará a ver cómo las personas de este estudio están conectadas entre sí. Por ejemplo, a través de amigos mutuos. También nos ayudará a recordar de quién hablamos hoy, la próxima vez que nos veamos. Recuerde que todo lo que me diga es confidencial y que protegeremos la privacidad de las personas que mencione. En ningún momento usaremos esta información para contactar personas.

Está bien nombrar a las personas con las que ha interactuado en los últimos 6 meses para más de una de las siguientes preguntas.

| VAR name | ITEM | PROBE: |
| --- | --- | --- |
| NGaTalk | 1. In the past 6 months, with whom did you talk to about the most private and personal things in your life?   En los últimos 6 meses, ¿con quién habló sobre las cosas más privadas y personales de su vida? | **Probe people they might trust:** Family, romantic partners, best friends, therapists, people of their faith/religious community, coworkers, others? |
| NGaHealth | 1. In the past 6 months, with whom did you talk to or ask for advice about your health, HIV, or PrEP?   En los últimos 6 meses, ¿con quién habló o pidió consejo sobre su salud, VIH o PrEP? | **Probe who they get health advice from:** providers, friends, family, coworkers, others? |
| NGaSex | 1. In the past 6 months, with whom did you have oral, anal, or vaginal sex that were 18 years of age or older? *Please tell me the names starting with your MOST recent sexual partner*.   En los últimos 6 meses, ¿con qué personas de 18 años o mayores tuvo sexo oral, anal, o vaginal? *Por favor, dígame los nombres comenzando con su pareja sexual MÁS reciente*. | **Probe sex partners for both romantic and physical attractions chronologically**; explore primary partners, friends, hook ups, app dates, others? |
| NGaDrug | 1. With whom did you drink alcohol, or use drugs more than once in the past 6 months?   En los últimos 6 meses, ¿con quién consumió alcohol o usó drogas más de una vez? | **Probe casual and heavy substance** use with friends, acquaintances, sexual partners, family, coworkers, others? |
| NGaHang | **Interviewers note: This can include people they regularly hang out with in group settings that they might not personally invite to hang out.**   1. In the past 6 months, with whom did you hang out or socialize with **regularly** in person or through technology? By regularly we mean you hang out typically several times a week (includes people you didn’t invite).   (En los últimos 6 meses, ¿con quién salió o socializó regularmente en persona o a través de la tecnología? Regularidad: referimos a que suele pasar el rato varias veces a la semana (incluye a quienes no invitó))  **Programming Note:** Character limitations in Network Canvas prohibit a direct translation of this item. The direct translation would be:  En los últimos 6 meses, ¿con quién salió o socializó regularmente en persona o a través de la tecnología? Con regularidad nos referimos a que suele pasar el rato varias veces a la semana (incluye personas a las que no invitó). | **Probe for both close and casual relationships** with friendships, family, coworkers, acquaintances, other couples, informal social groups, others?  **Interviewers note:** This can include people they regularly hang out with in group settings that they might not personally invite to hang out (e.g., your cousin’s friend that is always making rude comments about gay people). |
| NGaReject | **Interviewers note:** **probe for people who specifically don’t accept them because of their ethnic, masculine, or sexual identities.**   1. Who are people in your life that make comments (even as a joke) that make you think or feel like they don't accept you for who you are? This can include people that you don’t see very often.   ¿Quiénes son las personas en su vida que hacen comentarios (incluso de broma) que le hacen pensar o sentir que no le aceptan por lo que es? Esto puede incluir personas que no ve con mucha frecuencia. | **Explore tenuous or previously close relationships** with family, friends, coworkers, people of their faith/religious community, others?  Start by exploring if there are any people like this they have interacted with in the past 6 months, then explore the past year, then explore beyond 1 year. This includes people who make the participant feel this way even if the comments are not directed at them or they are not out to them (even if they are successful at avoiding them). |
| NGaAvoid | **Interviewers note: probe for people who specifically don’t accept them because of their ethnic, masculine, or sexual identities.**   1. Who are people in your life that you might want to avoid because they say or do things that make you feel uncomfortable or unwelcome? This can include people that you don’t see very often.   ¿Quiénes son las personas en su vida que quizás quiera evitar porque dicen o hacen cosas que le hacen sentir incómodo o no deseado? Esto puede incluir personas que no ve con mucha frecuencia. | **Explore challenging, hurtful, and obligatory relationships** with family members, friends of friends in their ‘friend group’, hook-ups/dates, dating app encounters, coworkers, neighbors, people of their faith/religion, others?  Start by exploring if there are any people like this they have interacted with in the past 6 months, then explore the past year, then explore beyond 1 year, especially if thinking about this person evokes an emotional response (even if they are successful at avoiding them) |

**If more than 5 alters are named, the following questions are asked at baseline:**

| If you named more than five people for each of the previous questions, I will ask you to only select the **TOP FIVE** who are most important to you in each category.  Si nombró a más de cinco personas para cada una de las preguntas anteriores, le pediré que solo seleccione las **CINCO MAS IMPORTANTES**. [select top five names from alter list Alter_1..n_] | | |
| --- | --- | --- |
| VAR name | ITEM | Response Option (value) |
| nTalk5 | For alters listed in Q3 (NGaTalk):   1. Of the people that you talk to about the most private and personal things in your life, which **5** are the **MOST important** to you?   De las personas con las que habla sobre las cosas más privadas y personales de su vida, ¿cuáles son las 5 MÁS importantes para usted? | **SELECT TOP 5 ONLY:**  Sociogram: (TRUE) |
| nHealth5 | For alters listed in Q4 (NGaHealth):   1. Of the people you talked to or asked for advice about your health, HIV, or PrEP, which **5** are the **MOST important** to you?   De las personas con las que habló o pidió consejos sobre su salud, el VIH o la PrEP, ¿cuáles son las 5 más importantes para usted? | **SELECT TOP 5 ONLY:**  Sociogram: (TRUE) |
| nSex5 | For alters listed in Q5 (NGaSex):   1. Of the people you had anal or vaginal sex with, which **5** are the **MOST important** to you (or do you see most often)?   De las personas con las que tuvo sexo anal o vaginal, ¿cuáles son las 5 MÁS importantes para usted (o las que ve con más frecuencia)? | **SELECT TOP 5 ONLY:**  Sociogram: (TRUE) |
| nDrug5 | For alters listed in Q6 (NGaDrug):   1. Of the people you drank alcohol, or used drugs with , which **5** are the **MOST important** to you (or do you see most often)?   De las personas con las que bebió alcohol o consumió drogas, ¿cuáles son las 5 MÁS importantes para usted (o las que ve con más frecuencia)? | **SELECT TOP 5 ONLY:**  Sociogram: (TRUE) |
| nHang5 | For alters listed in Q7 (NGaHang):   1. Of the people you hung out or socialized with regularly**,** which **5** are the **MOST important** to you (or do you see most often)?   De las personas con las que salió o socializó con regularidad, ¿cuáles son las 5 MÁS importantes para usted (o las que ve con más frecuencia)? | **SELECT TOP 5 ONLY:**  Sociogram: (TRUE) |
| nReject5 | For alters listed in Q7 (NGaReject):   1. Of the people who make comments (even as a joke) that make you think or feel like they don't accept you for who you are**,** which are the top **5** that **make you feel this way the MOST**?   De las personas que hacen comentarios (incluso de broma) que le hagan pensar o sentir que no le aceptan por lo que es, ¿cuáles son los 5 principales que le hacen sentir MÁS de esta manera? | SELECT TOP 5 ONLY:  Sociogram: (TRUE) |
| nAvoid5 | For alters listed in Q7 (NGaAvoid):   1. Of the people you might want to avoid because they say or do things that make you feel uncomfortable or unwelcome**,** which are the top **5** that **make you feel this way the MOST** ?   De las personas a las que quizás quiso evitar porque dicen o hacen cosas que le hacen sentir incómodo o no deseado, ¿cuáles son las 5 principales que le hacen sentir MÁS de esta manera? | SELECT TOP 5 ONLY:  Sociogram: (TRUE) |
