# Supplementary material for: A Novel Social Network Approach to Measure Intersectional Stigma Among Latino Men Who Have Sex With Men in San Diego, California (NEXUS): Protocol for a Longitudinal Cohort Study
Source: JMIR Res Protoc. 2026 Feb 27;15:e72334. doi: 10.2196/72334 (PMC12954697; doi:10.2196/72334)
Supplement: Multimedia Appendix 2 [file resprot-v15-e72334-s002.docx]

**NEXUS Longitudinal Social Network Data Integration Protocol**

At the time the NEXUS protocol was implemented, Network Canvas was not designed to support longitudinal social network data collection by retaining alters across Month 0 (M0), Month 6 (M6), and Month 12 (M12) social network interviews (SNI). To address this limitation, we developed a longitudinal social network data integration protocol for NEXUS described below. In brief this protocol is completed in three steps: (1) use SNI data from the previous interview to generate an Alter Report, (2) Enter data from the Alter Report ahead of scheduled interviews, and (3) Assess what alters left or entered the network over time.

# Step 1: Prepare Alter Reports for Follow Up Interviews

Step 1 uses SNI data from the previous interview to generate an Alter Report for the upcoming interview. Specifically, M0 Alter Reports are created for M6 interviews, and M6 Alter Reports are created for M12 interviews. If a participant did not complete the M6 interview, then the M0 Alter Report was used for the M12 interview. Note that M6 Alter Reports require additional steps to account for alters that left or newly entered the network at the M6 interview.

To generate Alter Reports, we used the following documents:

**Alter Report Log**. To generate Alter Reports, we recommend you keep a separate Alter Report log to track which Alter Reports have been done, and if there are any anomalies worth noting to the interviewer ahead of their follow up interview. For example, we found it helpful to identify if the participant did not report any alters for a specific name generator, or if there were an unusually high number.

**Alter Report Syntax for SPSS.** We utilized SPSS to generate our Alter Reports by identifying what name generators each alter was affiliated with and if that alter was also identified as a Top 5 alter for any of the name generators. Having the information on whether an alter is a Top 5 alter or not lets us know if detailed ISS data on that alter was collected in the previous interview, and whether or not an alter moves in and out of importance (i.e., Top 5) over time. Sample syntax below can be used to reconstruct Alter Reports in other statistical management software.

**Alter Report Template.** We kept a word document as a template to copy and paste the Alter Report from SPSS output into a more user-friendly document that could be securely accessed by the study field coordinator.

**Best Practices:**

1. Generate M0 Alter Reports monthly by Baseline (M0) interview month (e.g., May 2021, June 2021) to make them easier to locate when a participant is due for a M6 or a M12 interview as the baseline interview date will not change.
   - Generate one Alter Report at a time, to avoid mislabeling an Alter Report with the wrong participant ID (PID).
   - Add your initials to the Alter Report log once you have generated the report. Repeat this for each PID in the Alter Report Log for that month.
2. Copy the ‘M0 .CSV’ or ‘M6 .CSV’ ‘attributes’ file that is exported from Network Canvas from a specific participant interview to a working folder. Do not move the original data file, only work from a copy of that file to ensure data integrity.
3. Open the .CSV file from the working folder and save as an Excel Workbook or Excel 97-2003 Workbook (.xls) to avoid errors in SPSS reading in the data. We ran into problems importing the .CSV files into SPSS and found the .xls files to be consistently reliable.
   - NOTE: if you use a value for missing data (e.g. 9999) on an alter identifier you are using to generate the Alter Report, such as their name, you may need to reformat that column in the excel file to be text format so that the data are read in by SPSS correctly.
4. Open SPSS and import ‘M0 .xls’ or M6 . xls’ ‘attributes’ file into SPSS.
5. Open your M0 syntax or your M6 syntax file. Run the SYNAX file to generate each individual participant’s M0 or M6 Alter Report.
   - Open M0 or M6 Alter Report TEMPLATE.
   - Copy the Alter Report Table from the SPSS output to the template and add PID and Notes as needed.
   - Save the Alter Report word document to a secure study file to be entered into Network Canvas by two study team members (e.g., field coordinator and interviewer) ahead of scheduled M6 or M12 interviews.
6. Clean the workspace before running a new Alter Report.
   - Delete the copy of the .CSV and .xls files in the working folder AND your computer’s recycle bin.
   - Close the SPSS file without saving it.
   - Complete your entry in the Alter Report log.

## **SYNTAX Example: M0 Alter Report**

*****STEP A:** COMPUTE M0TOP5 (numeric) VAR (1=TRUE, MISSING = not a top 5 alter)

Run syntax, review frequencies of responses by Top5 alter type are recoded as a M0Top5 alter

**IF** nTalk5=1 OR nHealth5=1 OR nSex5=1 OR nDrug5=1 OR nHang5=1 OR nReject5=1 OR nAvoid5=1 M0Top5=1.

**EXECUTE**.

**LIST VARIABLES** = nTalk5 nHealth5 nSex5 nDrug5 nHang5 nReject5 nAvoid5 M0Top5.

*****STEP B:** RUN PARTICIPANT-SPECIFIC M0 ALTER REPORT (GROUPING = nodeID)

Run syntax, review table, confirm data appear in the following order:

nodeID, Name, NGaTalk NGaHealth NGaSex NGaDrug NGaHang NGaReject NGaAvoid M0Top5

**SUMMARIZE**

/TABLES=Name NGaTalk NGaHealth NGaSex NGaDrug NGaHang NGaReject NGaAvoid M0Top5

BY nodeID

/FORMAT=VALIDLIST NOCASENUM TOTAL LIMIT=2000

/TITLE='M0 Alter Report'

/MISSING=VARIABLE

/CELLS=NONE.

## **SYNTAX Example: M6 Alter Report**

****STEP A:** COMPUTE M6TOP5 (numeric) VAR (1=TRUE, MISSING=not a top 5 alter)

Run syntax, review frequencies of responses by Top5 alter type are recoded as a M6Top5 alter

**IF** NTalk56=1 OR nHealth56=1 OR nSex56=1 OR nDrug56=1 OR nHang56=1 OR nReject56=1 OR nAvoid56=1 M6Top5=1.

**EXECUTE**.

**LIST VARIABLES** = NTalk56 nHealth56 nSex56 nDrug56 nHang56 nReject56 nAvoid56 M6Top5.

*****STEP B:** COMPUTE Name Generator Variables that COLAPSE M0 alter that was retained (=1) AND M6 alters that are new to the network (=1) INTO A SINGLE VARIABLE

**IF** nTalkReta6_1=1 OR NGaTalk6=1 M6NGaTalk=1.

**IF** nHealthReta6_1=1 OR NGaHealth6=1 M6NGaHealth=1.

**IF** nSexReta6_1=1 OR NGaSex6=1 M6NGaSex=1.

**IF** nDrugReta6_1=1 OR NGaDrug6=1 M6NGaDrug=1.

**IF** nHangRela6_1=1 OR NGaHang6=1 M6NGaHang=1.

**IF** nRejectReta6_1=1 OR NGaReject6=1 M6NGaReject=1.

**IF** nAvoidReta6_1=1 OR NGaAvoid6=1 M6NGaAvoid=1.

**EXECUTE**.

**VALUE LABELS**

M6NGaTalk M6NGaHealth M6NGaSex M6NGaDrug M6NGaHang M6NGaReject M6NGaAvoid

1 TRUE.

**EXECUTE**.

*****STEP C:** COMPUTE NOTretM0 (M0 alters that were NOT Retained or left the network at M6 follow up)

run a frequency command to confirm number of M0 alters that were not retained

**IF** MISSING(M6NGaTalk) AND MISSING(M6NGaHealth) AND MISSING(M6NGaSex) AND MISSING(M6NGaDrug) AND MISSING(M6NGaHang) AND MISSING(M6NGaReject) AND MISSING(M6NGaAvoid) NOTretM0=1.

**EXECUTE**.

**VALUE LABELS**

NOTretM0

1 M0 Alter NOT Retained.

**EXECUTE**.

**FREQUENCIES** NOTretM0.

*****STEP D:** FILTER OUT NOTretM0 ~=1 ( i.e., M0 alters that were NOT Retained) before running the report

run Freq var on NOTretM0, the number of missing should equal the same number of missing in Step C

**USE** ALL.

**COMPUTE** filter_$=(MISSING(NOTretM0)).

**VARIABLE** **LABELS** filter_$ 'MISSING(NOTretM0) (FILTER)'.

**VALUE LABELS** filter_$ 0 'Not Selected' 1 'Selected'.

**FORMATS** filter_$ (f1.0).

**FILTER** BY filter_$.

**EXECUTE**.

**FREQUENCIES** NOTretM0.

*****STEP E:** RUN PARTICIPANT-SPECIFIC M6 ALTER REPORT (GROUPING = nodeID)

Run syntax, review table, confirm data appear in the following order:

nodeID Name NGaTalk NGaHealth NGaSex NGaDrug NGaHang NGaRejet NGaAvoid M0Top5

**SUMMARIZE**

/TABLES=Name M6NGaTalk M6NGaHealth M6NGaSex M6NGaDrug M6NGaHang M6NGaReject M6NGaAvoid M6Top5

BY nodeID

/FORMAT=VALIDLIST NOCASENUM TOTAL LIMIT=2000

/TITLE='M6 Alter Report'

/MISSING=VARIABLE

/CELLS=NONE.

# Step 2: Enter Alter Reports for Scheduled Follow Up Interviews

**Best Practices:**

1. Set up weekly Alter Report meetings with interviewers to read in the Alter Reports for all upcoming M6 and M12 interviews that have been scheduled for the coming week. This reduces the need for last minute Alter Report read-ins and ensures the interviewer has the data needed ahead of time.
2. Alter Report meetings consisted of our NEXUS field coordinator and the study interviewer assigned to a specific M6 or M12 interview. Using two individuals for this process helps to ensure data integrity and guard against data entry errors.
3. For each Alter Report, start a new M6 or M12 interview in Network Canvas. Select the correct M6 or M12 interview protocol for an English or Spanish interview, then scroll down to the section that says “Start a New Interview” on the home page.
4. The interviewer will confirm the participant ID (PID) for the scheduled M6 or M12 interview as they enter it into Network Canvas, and the field coordinator will confirm the correct PID on the Alter Report before proceeding.
5. For M6 or M12 interviews with confirmed PIDs, the interviewer will then navigate to enter new alters into the Network Canvas interview protocol.
   1. The field coordinator will read out the spelling of the alter characteristics that are entered (e.g., name) and which of the 7 name generators the alter is affiliated with and whether or not the alter was a Top 5 alter.
   2. The interviewer enters this information and then reads the entry back to the field coordinator to confirm all data was entered correctly and hits SAVE before proceeding to the next alter (see Figure 1).

**FIGURE 1:**


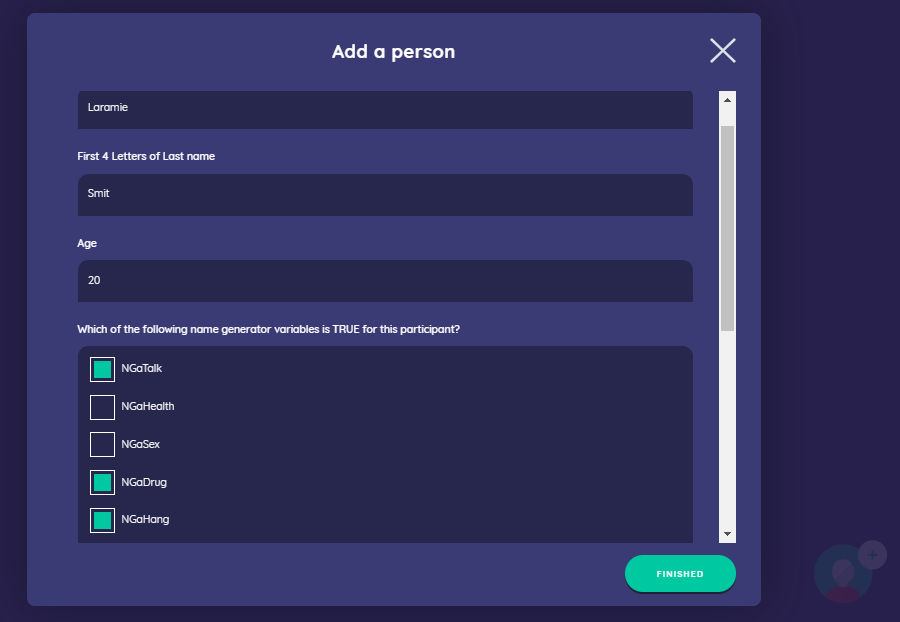


1. Once all alters have been entered, confirm that the total number of alters in the M6 or M12 SNI matches the number of alters in the Alter Report before closing out the interview by reading off the alter names/or alter IDs from Network Canvas to the field coordinator to double check all alters in the alter report are accounted for. We built this into the pre-interview component of the M6 and M12 SNI protocol as a reminder to complete this task and confirm that it had been completed.

| VAR name | ITEM | Response Option (value) |
| --- | --- | --- |
| aConfirm | 1. Did you enter the SAME number of alters listed on the M0 Alter Report and confirm all data are entered correctly? | Yes (1)  No, I need to come back and confirm the data (0) |

***Tip for M6 Alter Reports:*** *The M6 Alter Report will have removed any alters that were identified during the M0 interview but were NOT identified at the M6 interview. This means that the numbering on the left-hand side of the M6 alter report may skip numbers. (i.e., 1,3,5,6,9). You will need to count the number of alters to get an accurate number rather than looking at the numbering on the left of the Alter Report.*

1. ‘SAVE’ interview to finish later with the Participant (see Figure 2).

**FIGURE 2:**


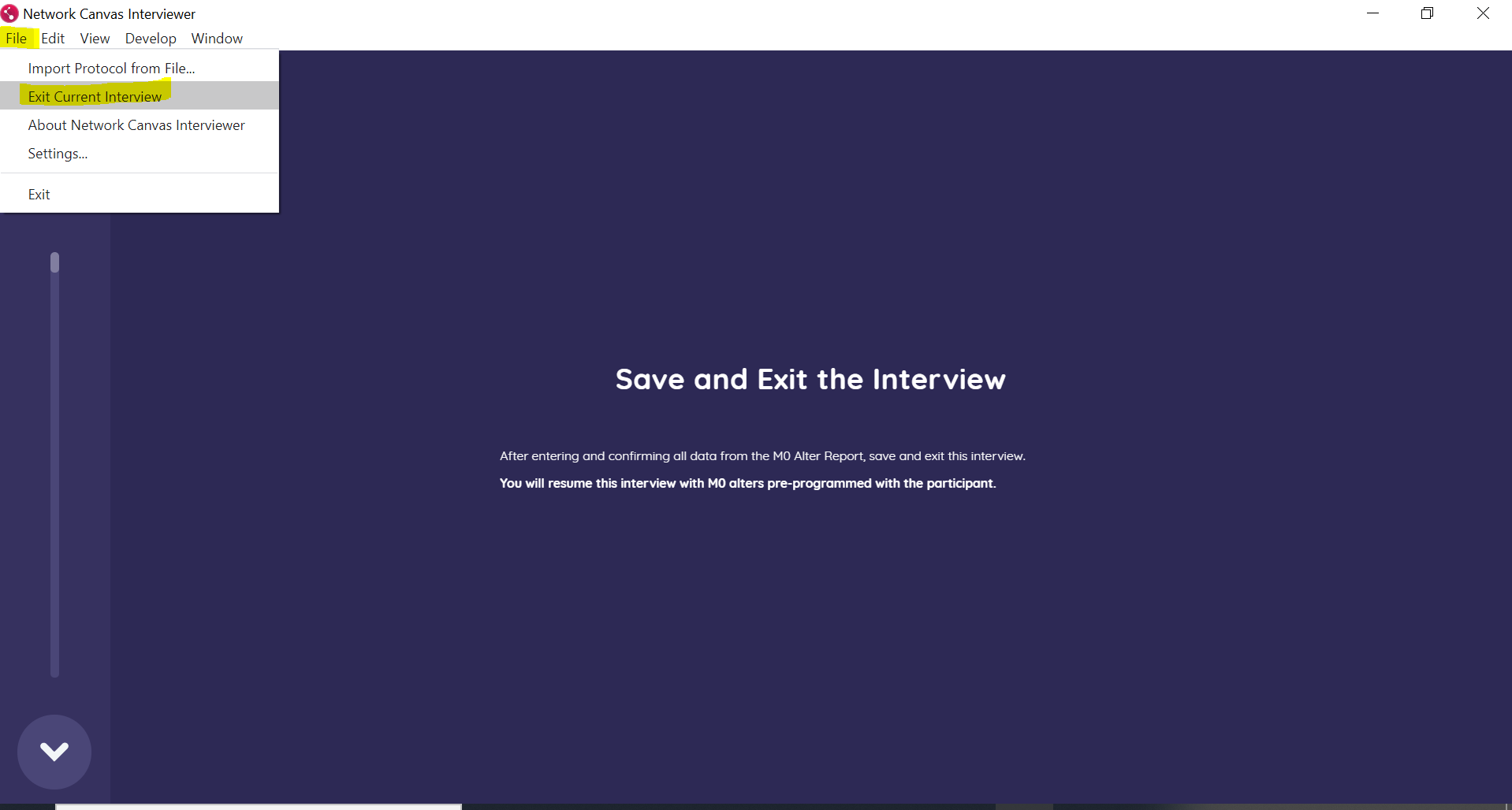


# Step 3: Identify Alters that Left/Entered the Network at Follow Up

**Best Practices:**

1. At the start of the M6/M12 interview, interviewers review all alters that were named or retained in the previous interview with the participant. Interviewer instructions and variables we programed into Network Canvas to capture this data in our M6 interview are provided as an example below:

**SNI 1: M0 NETWORK STABILITY (Month 6, all M0 alters)**

**READ:** Welcome back to NEXUS

Today we would like to check back in on the people we talked about at your last interview.

To make this easier, we already entered the names of those people just before your interview.

If you do not remember someone you told us about last time that is OK, you will be able to document this. Your answers will help us learn how changes in who you interact with might affect health promotion efforts like HIV prevention.

Please remember that everything you tell me is *confidential* and that we will protect the privacy of the people you mention. We will never at any time use this information to contact people.

**LEE:** Bienvenidos de Nuevo a NEXUS

Hoy nos gustaría volver a mencionar a las personas de las que hablamos en su última entrevista. Para facilitarlo, ya hemos introducido a este questionario los nombres de esas personas justo antes de esta entrevista.

Sus respuestas nos ayudarán a saber cómo los cambios en las personas con las que se relaciona pueden afectar sus esfuerzos de promoción de la salud, como la prevención del VIH.

Por favor, recuerde que todo lo que me diga es *confidencial* y que protegeremos la privacidad de las personas que mencione. En ningún momento utilizaremos esta información para podernos en contacto con las personas.

**M0 NETWORK STABILITY**

| **Programming Note:** List all alters entered from the M0 Alter Report. | | |
| --- | --- | --- |
| VAR name | ITEM | Response Option (value) |
| nStabilty_M6 | 1. How has your relationship changed with each of these people since your last interview?   ¿Cómo ha cambiado su relación con cada una de estas personas desde su última entrevista? | No interaction/ No hay interacción (0)  Less interaction/ Menos interacción (1)  Same interaction/ La misma interacción (2)  More interaction / Más interacción (3) |
| **Programming Note:** Only list aNGReject and aNGAvoid alters entered from the M0 Alter Report. | | |
| nConflict_M6 | 1. Which of these people have said or done things in the past that still make you feel like they don't accept you or make you feel uncomfortable or unwelcome. This can include people that you don't see very often.   ¿Cuáles de estas personas han dicho o hecho cosas en el pasado que todavía le hacen sentir que no le aceptan o le hacen sentir incómodo o no bienvenido? Esto puede incluir a personas a las que no ve muy a menudo. | I still feel this way/ Todavía me siento así (1)  I no longer feel this way / Ya no me siento así (2) |
| **REASONS FOR NOT INTERACTING WITH M0 ALTERS**  **Programming Note:** Only list alters that were listed as (a) No interaction for nStability_M6 unless they were also listed as (1) I still feel this way for nConflict_M6 | | |
| **No Interaction Past 6 Months / No ha interactuado en los últimos 6 meses**  **READ:**  We want to know a little bit more about why you have not interacted with some of the people you mentioned last time.  Queremos saber un poco más sobre por qué no ha interactuado con algunas de las personas que mencionó la última vez. | | |
| nLeft_M6 | 1. Which of the following reasons **BEST** describes why you have not interacted with this person in the past 6 months?   ¿Cuál de las siguientes razones describe MEJOR la razón por la que no ha interactuado con esta persona en los últimos 6 meses? | Our relationship ended/We stopped seeing each other/ Nuestra relación terminó/dejamos de vernos (1)  They were a one-time sexual partner/ Fueron una pareja sexual de una sola vez (2)  Change in where I/they worked, worshiped, or hung out/ Cambio el lugar donde yo/ellos trabajaban, religión o reunión (3)  Change in where I/they went for health or professional services./ Cambio el lugar al que yo/ellos acudían para recibir cuidado médico o servicios profesionales (4)  I/they moved/ Yo/ellos se mudaron (5)  They died/ Murieron (6)  They ghosted me/started avoiding me/ Me ignoraron/dejaron a un lado/empezaron a evitarme (7)  We had a disagreement or falling out/ Tuvimos un desacuerdo o una pelea (8)  I don’t remember this person/ No recuerdo a esta persona. (9)  Other (specify below)/ Otro (especifique) (10) |
| nLeft_OM6 | 1. Please specify the other reason you have not interacted with this person.   Por favor, especifique la otra razón por la que no ha interactuado con esta persona. | *[ TEXT FIELD ]* |
| nLeft_RM6 | 1. You have not interacted with the following people in the past 6 months because they either started ghosting/avoiding you or you had a disagreement/falling out. Was this change in your relationship because of your...   No se ha relacionado con las siguientes personas en los últimos 6 meses porque lo han ignorado/evitado o ha tenido un desacuerdo/pelea con ellos. ¿Este cambio en su relación se debe a su…..? | Ethnicity/ Origen étnico(1)  Masculine expression/ Expresión masculina (2)  Sexuality/ Sexualidad (3)  Something else/ Otra cosa |
| NLeft_ORM6 | 1. Briefly, why do you believe they started ghosting/avoiding you or you had a disagreement/falling out?   Brevemente, ¿por qué crees que lo han ignorado/evitado o ha tenido un desacuerdo/pelea con ellos? | *[ TEXT FIELD ]* |
| **Programming Note:** List all M0 alters not listed in nLeft_M6 – this includes M0 alters that participant interacted with (nStability_M6 >0) AND people who said/did something that still bothers them (nConflict_M6 = 1) | | |

1. To move forward with M0 Alters that had not left the network at follow up and retain them in their proper name generator categories, we had to program Network Canvas to list all alters that were still relevant to the participant’s network and move them into alter name generator specific categories where they would later join new alters named for each name generator when it was time to select the TOP 5 names per name generator at follow up.

| **Please Stop Here**  Please stop here while the interviewer programs the survey to optimize your experience.  **Por favor espere aqui**  Espere aquí mientras el entrevistador programa la encuesta para optimizar su experiencia.  ***Interviewer Note:*** *Let the participant know that you will put the names of people they have interacted with in a holding bin so you can talk about your interactions with them later in the interview.*  *Quickly place all alters in their respective bins.* | | |
| --- | --- | --- |
| VAR name | ITEM | Response Option (value) |
| nTalkReta6 | **Interviewer Instructions:** Place all alters in the Talk about Private & Personal Things bin. | Talk about Private & Personal Things (1)  Do not place alters here (0) |
| nHealthReta6 | **Interviewer Instructions:** Place all alters in the Talked to about health advice bin. | Talked to about health advice (1)  Do not place alters here (0) |
| nSexReta6 | **Interviewer Instructions:** Place all alters in the Sexual Partner bin. | Sexual Partner (1)  Do not place alters here (0) |
| nDrugReta6 | **Interviewer Instructions:** Place all alters in the Drank Alcohol or Used Drugs bin. | Drank Alcohol or Used Drugs (1)  Do not place alters here (0) |
| nHangReta6 | **Interviewer Instructions:** Place all alters in the Hangout regularly bin. | Hangout regularly (1)  Do not place alters here (0) |
| nRejectReta6 | **Interviewer Instructions:** Place all alters in the Don't Accept you bin. | Don't Accept you (1)  Do not place alters here (unless deceased) (0) |
| nAvoidReta6 | **Interviewer Instructions:** Place all alters in the You want to Avoid bin. | You want to Avoid (1)  Do not place alters here (unless deceased) (0) |

1. We identified the need to remove alters from the next stages of follow up interviews because alters may have left the network because they were no longer living; however, they were a conflictual tie. We removed these alters, so that they would not continue to show up throughout the follow up interview which could cause undue stress. To address these noted instructions in the two response categories for conflictual ties, we indicated where those alters who were deceased should be classified to keep them from moving forward into the rest of the interview.
2. Next, we reinstituted our seven name generators to identify who had entered the network at follow up. See Appendix 1 for specific name generator items. Our instructions to elicit NEW alters at follow up is provided below as an example.

**SNI 2: NEW ALTERS NAME GENERATORS (Months 6)**

**Now let's talk about new interactions since we last talked.**

**Ahora hablaremos sobre personas nuevas con las que ha interactuado en los últimos 6 meses**

**READ:** Now let’s see if there are any new adults who are 18 years or older who you interacted with in the **past 6 months** **that you did not name in your last interview.** These can be adults you already knew or recently met that you interacted with in-person, by phone or text, or through social media or dating apps. This includes adults who are family, friends, sexual or romantic partners. It can also include other adults like co-workers, neighbors, healthcare providers or people from your religious group.

Remember, having names and demographic information of the people you interacted with will help us see how people in this study are connected to one another. For example, through mutual friends. It will also help us remember who we talked about today, the next time we meet. Please remember that everything you tell me is confidential and that we will protect the privacy of the people you mention. We will never at any time use this information to contact people.

You can name the same person for more than once.

**LEE:** Ahora veamos si hay algún nuevo adulto de 18 años o más con el que haya interactuado en los últimos 6 meses y que no haya nombrado en su última entrevista. Estos pueden ser adultos con los que interactuó en persona, por teléfono o mensaje de texto, o a través de las redes sociales o aplicaciones de citas. Esto incluye a los adultos que son familiares, amigos, parejas sexuales o románticas. También puede incluir a otros adultos como compañeros de trabajo, vecinos, proveedores de atención médica o personas de su grupo religioso.

Recuerde que tener los nombres y la información demográfica de las personas con las que ha interactuado nos ayudará a ver cómo las personas de este estudio están conectadas entre sí. Por ejemplo, a través de amigos mutuos. También nos ayudará a recordar de quién hablamos hoy, la próxima vez que nos veamos. Por favor recuerde que todo lo que me diga es confidencial y que protegeremos la privacidad de las personas que mencione. En ningún momento usaremos esta información para contactar personas.

Está bien nombrar a las personas con las que ha interactuado en los últimos 6 meses para más de una de las siguientes preguntas.
