# Supplementary material for: A Novel Social Network Approach to Measure Intersectional Stigma Among Latino Men Who Have Sex With Men in San Diego, California (NEXUS): Protocol for a Longitudinal Cohort Study
Source: JMIR Res Protoc. 2026 Feb 27;15:e72334. doi: 10.2196/72334 (PMC12954697; doi:10.2196/72334)
Supplement: Multimedia Appendix 3 [file resprot-v15-e72334-s003.docx]

NEXUS Social Network Intersectional Stigma Questions

# BASELINE

NOTE: Based on piloting NEXUS measures, we asked 9 additional questions in between the anticipated and enacted ISS exposure items to help participants draw a distinction between future and past experiences and reduce perceptions that the items were repetitive. These items reflected whether alters actively made participants (1) feel welcome or (2) affirmed their ethnicity, masculinity, or sexuality, and (3) whether participants avoided specific alters because of comments they made about their ethnicity, masculinity, or sexuality.

**ANTICIPATED ISS EXPOSURE (Month 0: ALL top 5 alters)**

**ANTICIPATED ISS EXPOSURE FROM ALTERS**

| **Programming Note:**  For section, list all **TOP 5** alters named for any of the 7 name generator questions. | | |
| --- | --- | --- |
| **The next 9 questions ask about how these people might treat you differently in the FUTURE because of your ethnicity, masculinity, and sexuality.**  **Las siguientes 9 preguntas se refieren a cómo estas personas podrían tratarlo de manera diferente en el FUTURO debido a su origen étnico, masculinidad y sexualidad.** | | |
| VAR name | ITEM | Response Option (value) |
| aANTstereoL | 1. In the **FUTURE**, how likely is it that these people will make negative comments or jokes about Latinos that bother you?   En el FUTURO, ¿qué tan probable es que estas personas hagan comentarios negativos o bromas sobre los(as) latinos(as) que le molesten? | Very unlikely/ muy improbable (1)  Unlikely/ improbable (2)  it’s possible/ Es posible (3)  Likely/ probable (4)  Very likely/ Muy probable (5) |
| aANTstereoM | 1. In the **FUTURE**, how likely is it that these people will make negative comments or jokes about men who don’t act ‘traditionally’ masculine that bother you?   En el FUTURO, ¿qué tan probable es que estas personas hagan comentarios negativos o bromas sobre hombres que no actúan "tradicionalmente" masculinos que le molesten? | Very unlikely/ muy improbable (1)  Unlikely/ improbable (2)  it’s possible/ Es posible (3)  Likely/ probable (4)  Very likely/ Muy probable (5) |
| aANTstereoG | 1. In the **FUTURE**, how likely is it that these people will make negative comments or jokes about men who have sex with other men that bother you?   En el FUTURO, ¿qué probabilidades hay de que estas personas hagan comentarios negativos o bromas sobre hombres que tienen relaciones sexuales con otros hombres que le molesten? | Very unlikely/ muy improbable (1)  Unlikely/ improbable (2)  it’s possible/ Es posible (3)  Likely/ probable (4)  Very likely/ Muy probable (5) |
| **People who would judge you or look down on you in the future…**  **Personas que le juzgarían o menospreciarían en el futuro...** | | |
| aANTdownL | 1. In the **FUTURE**, how likely is it that these people will judge you or look down on you because you are Latino?   En el FUTURO, ¿qué probabilidades hay de que estas personas le juzguen o le menosprecien por ser latino? | Very unlikely/ muy improbable (1)  Unlikely/ improbable (2)  it’s possible/ Es posible (3)  Likely/ probable (4)  Very likely/ Muy probable (5) |
| aANTdownM | 1. In the **FUTURE**, how likely is it that these people will judge you or look down on you because you don’t act ‘traditionally’ masculine?   En el FUTURO, ¿qué probabilidades hay de que estas personas le juzguen o le menosprecien porque no actúa "tradicionalmente" masculino? | Very unlikely/ muy improbable (1)  Unlikely/ improbable (2)  it’s possible/ Es posible (3)  Likely/ probable (4)  Very likely/ Muy probable (5) |
| aANTdownG | 1. In the **FUTURE**, how likely is it that these people will judge you or look down on you because you have sex with other men?   En el FUTURO, ¿qué probabilidades hay de que estas personas le juzguen o le menosprecien por tener relaciones sexuales con otros hombres? | Very unlikely/ muy improbable (1)  Unlikely/ improbable (2)  it’s possible/ Es posible (3)  Likely/ probable (4)  Very likely/ Muy probable (5) |
| **People who would treat you poorly or avoid you in the future…**  **Personas que le tratarían mal o le evitarían en el futuro...** | | |
| aANTavoidL | 1. In the **FUTURE**, how likely is it that these people will treat you poorly or avoid you because you are Latino?   En el FUTURO, ¿qué probabilidades hay de que estas personas le traten mal o le eviten por ser latino? | Very unlikely/ muy improbable (1)  Unlikely/ improbable (2)  it’s possible/ Es posible (3)  Likely/ probable (4)  Very likely/ Muy probable (5) |
| aANTavoidM | 1. In the **FUTURE**, how likely is it that these people will treat you poorly or avoid you because you don’t act ‘traditionally’ masculine?   En el FUTURO, ¿qué probabilidades hay de que estas personas le traten mal o le eviten porque no actúa "tradicionalmente" masculino? | Very unlikely/ muy improbable (1)  Unlikely/ improbable (2)  it’s possible/ Es posible (3)  Likely/ probable (4)  Very likely/ Muy probable (5) |
| aANTavoidG | 1. In the **FUTURE**, how likely is it that these people will treat you poorly or avoid you because you have sex with other men?   En el FUTURO, ¿qué probabilidades hay de que estas personas le traten mal o le eviten porque tiene relaciones sexuales con otros hombres? | Very unlikely/ muy improbable (1)  Unlikely/ improbable (2)  it’s possible/ Es posible (3)  Likely/ probable (4)  Very likely/ Muy probable (5) |

**ISS AVOIDANCE (Month 0: ALL top 5 alters)**

**EGO AVOIDANCE OF ALTERS DUE TO ISS**

| **The next 3 questions ask whether YOU have ever wanted to avoid or not interact with any of these people because of their views towards Latinos, masculinity, or sexuality.**  **Las siguientes 3 preguntas preguntan si USTED alguna vez ha querido evitar o no interactuar con alguna de estas personas debido a sus puntos de vista hacia los latinos, la masculinidad o la sexualidad.** | | |
| --- | --- | --- |
| VAR name | ITEM | Response Option (value) |
| EgoAvoidL | 1. Which of these people have **YOU** wanted to **avoid or not interact** **with** because of their views on Latinos?   ¿Con cuál de estas personas ha querido evitar o no interactuar debido a sus opiniones sobre los(as) latinos(as)? | Never/ Nunca (0)  Yes, more than 6 months ago/ Sí, hace MÁS de 6 meses (1)  Yes, in the past 6 months/ Sí, en los ÚLTIMOS 6 meses (2) |
| EgoAvoidM | 1. Which of these people have **YOU** ever wanted to **avoid or not interact** **with** because their views on how you express your masculinity?   ¿Con cuál de estas personas ha querido evitar alguna vez o no interactuar debido a sus puntos de vista sobre cómo expresa su masculinidad? | Never/ Nunca (0)  Yes, more than 6 months ago/ Sí, hace más de 6 meses (1)  Yes, in the past 6 months/ Sí, en los últimos 6 meses (2) |
| EgoAvoidG | 1. Which of these people have **YOU** ever wanted to **avoid or not interact** **with** because of their views on men who have sex with other men?   ¿Con cuál de estas personas ha querido evitar o no interactuar debido a las opiniones que tienen sobre los hombres que tienen sexo con otros hombres? | Never/ Nunca (0)  Yes, more than 6 months ago/ Sí, hace más de 6 meses (1)  Yes, in the past 6 months/ Sí, en los últimos 6 meses (2) |

**ENACTED ISS EXPOSURE (Month 0: ALL top 5 alters)**

**ENACTED ISS EXPOSURE FROM ALTERS**

| **Programming Note:**  For section, list all **TOP 5** alters named for any of the 7 name generator questions. |
| --- |

| **Now, we want to ask 9 questions, similar to the ones we asked previously. These questions will ask how often these people have treated you differently in the PAST because of your ethnicity, masculinity, and sexuality.**  **Ahora, queremos hacer 9 preguntas similares a las que hicimos anteriormente. Estas preguntas serán sobre la frecuencia en la que estas personas le han tratado de manera diferente en el PASADO debido a su origen étnico, masculinidad y sexualidad.** | | |
| --- | --- | --- |
| VAR name | ITEM | Response Option (value) |
| aENTstereoL | 1. In the **PAST**, how often has each of these people made negative comments or jokes about Latinos that bothered you?   En el PASADO, ¿con qué frecuencia cada una de estas personas hizo comentarios negativos o bromas sobre los latinos que le molestaron? | Never/ Nunca (1)  Not Often/ raramente (2)  Somewhat Often/ algunas veces (3)  Often/ frecuentemente (4)  Very Often/ muy frecuentemente (5) |
| aENTstereoM | 1. In the **PAST**, how often has each of these people made negative comments or jokes about men who don’t act ‘traditionally’ masculine that bothered you?   En el PASADO, ¿con qué frecuencia cada una de estas personas ha hecho comentarios negativos o bromas sobre hombres que no actúan "tradicionalmente" masculinos que le molestaban? | Never/ Nunca (1)  Not Often/ raramente (2)  Somewhat Often/ algunas veces (3)  Often/ frecuentemente (4)  Very Often/ muy frecuentemente (5) |
| aENTstereoG | 1. In the **PAST**, how often has each of these people made negative comments or jokes about men who have sex with other men that bothered you?   En el PASADO, ¿con qué frecuencia cada una de estas personas ha hecho comentarios negativos o bromas sobre hombres que tienen relaciones sexuales con otros hombres que le molestaban? | Never/ Nunca (1)  Not Often/ raramente (2)  Somewhat Often/ algunas veces (3)  Often/ frecuentemente (4)  Very Often/ muy frecuentemente (5) |
| **People who judged you or look down on you in the past…**  **Personas que le juzgaron o menospreciaron en el pasado ...** | | |
| aENTdownL | 1. In the **PAST**, how often has each of these people judged you or looked down on you because you are Latino?   En el PASADO, ¿con qué frecuencia cada una de estas personas le ha juzgado o despreciado por ser latino? | Never/ Nunca (1)  Not Often/ raramente (2)  Somewhat Often/ algunas veces (3)  Often/ frecuentemente (4)  Very Often/ muy frecuentemente (5) |
| aENTdownM | 1. In the **PAST**, how often has each of these people judged you or looked down on you because you don’t act ‘traditionally’ masculine?   En el PASADO, ¿con qué frecuencia cada una de estas personas le ha juzgado o despreciado porque no actúa "tradicionalmente" masculino? | Never/ Nunca (1)  Not Often/ raramente (2)  Somewhat Often/ algunas veces (3)  Often/ frecuentemente (4)  Very Often/ muy frecuentemente (5) |
| aENTdownG | 1. In the **PAST**, how often has each of these people judged you or looked down on you because you have sex with other men?   En el PASADO, ¿con qué frecuencia cada una de estas personas le ha juzgado o despreciado por tener relaciones sexuales con otros hombres? | Never/ Nunca (1)  Not Often/ raramente (2)  Somewhat Often/ algunas veces (3)  Often/ frecuentemente (4)  Very Often/ muy frecuentemente (5) |
| **People who treated you poorly or avoided you in the past…**  **Personas que le trataron mal o le evitaron en el pasado ...** | | |
| aENTavoidL | 1. In the **PAST**, how often has each of these people treated you poorly or avoided you because you are Latino?   En el PASADO, ¿con qué frecuencia cada una de estas personas le trató mal o le evitó por ser latino? | Never/ Nunca (1)  Not Often/ raramente (2)  Somewhat Often/ algunas veces (3)  Often/ frecuentemente (4)  Very Often/ muy frecuentemente (5) |
| aENTavoidM | 1. In the **PAST** how often has each of these people treated you poorly or avoided you because you don’t act ‘traditionally’ masculine?   En el PASADO, ¿con qué frecuencia cada una de estas personas le trató mal o le evitó porque no actúa "tradicionalmente" masculino? | Never/ Nunca (1)  Not Often/ raramente (2)  Somewhat Often/ algunas veces (3)  Often/ frecuentemente (4)  Very Often/ muy frecuentemente (5) |
| aENTavoidG | 1. In the **PAST**, how often has each of these people treated you poorly or avoided you because you have sex with other men?   En el PASADO, ¿con qué frecuencia cada una de estas personas lo trató mal o lo evitó porque tuvo relaciones sexuales con otros hombres? | Never/ Nunca (1)  Not Often/ raramente (2)  Somewhat Often/ algunas veces (3)  Often/ frecuentemente (4)  Very Often/ muy frecuentemente (5) |

**INTERSECTIONAL SUPPORT (Month 0: ALL top 5 alters)**

**INTERSECTIONAL AFFIRMATION AND SUPPORT FROM ALTERS**

| **The questions from this section ask about positive experiences you have had with these people related to your ethnicity, masculinity, and sexuality.**  **Las preguntas de esta sección se refieren a experiencias positivas que ha tenido con estas personas relacionadas con su origen étnico, masculinidad y sexualidad.** | | |
| --- | --- | --- |
| VAR name | ITEM | Response Option (value) |
| aAffirmL | 1. Which of these people have made you think or believe that they support your Latino identity? *This could be verbal or non-verbal gestures of support.*   ¿Cuáles de estas personas le han hecho pensar o creer que apoyan su identidad latina? *Pueden ser gestos de apoyo verbales o no verbales.* | Never/ Nunca (0)  Yes, more than 6 months ago/ Sí, hace MÁS de 6 Meses (1)  Yes, in the past 6 months/ Sí, en los ÚLTIMOS 6 Meses (2) |
| aAffirmM | 1. Which of these people have made you think or believe that they support how you express your masculinity? *This could be verbal or non-verbal gestures of support.*   ¿Cuáles de estas personas le han hecho pensar o creer que apoyan la forma en que expresa su masculinidad? *Pueden ser gestos de apoyo verbales o no verbales*. | Never/ Nunca (0)  Yes, more than 6 months ago/ Sí, hace MÁS de 6 Meses (1)  Yes, in the past 6 months/ Sí, en los ÚLTIMOS 6 Meses (2) |
| aAffirmG | 1. Which of these people have made you think or believe that they support you as a man who has sex with other men? *This could be verbal or non-verbal gestures of support.*   ¿Cuáles de estas personas le han hecho pensar o creer que le apoyan como hombre que tiene sexo con otros hombres? *Pueden ser gestos de apoyo verbales o no verbales*. | Never/ Nunca (0)  Yes, more than 6 months ago/ Sí, hace MÁS de 6 Meses (1)  Yes, in the past 6 months/ Sí, en los ÚLTIMOS 6 Meses (2) |
| **People who made you feel welcomed or embraced…**  **Personas que le hicieron sentir bienvenido o aceptado ...** | | |
| aWelcomeL | 1. Which of these people have **made you feel welcome or embraced** your experiences as someone who is Latino?   ¿Cuál de estas personas le ha **hecho** **sentir bienvenido o aceptado** sus experiencias como **latino**? | Never/ Nunca (0)  Yes, more than 6 months ago/ Sí, hace MÁS de 6 Meses (1)  Yes, in the past 6 months/ Sí, en los ÚLTIMOS 6 Meses (2) |
| aWelcomeM | 1. Which of these people have **made you feel welcome or embraced** how you express your masculinity?   ¿Cuál de estas personas le ha hecho sentir bienvenido o aceptado cómo expresa su masculinidad? | Never/ Nunca (0)  Yes, more than 6 months ago/ Sí, hace MÁS de 6 Meses (1)  Yes, in the past 6 months/ Sí, en los últimos 6 meses (2) |
| aWelcomeG | 1. Which of these people have **made you feel welcome or embraced** your experiences as a man who has sex with other men?   ¿Cuál de estas personas le ha hecho sentir bienvenido o aceptado la manera cómo expresa experiencias como hombre que tiene sexo con otros hombres? | Never/ Nunca (0)  Yes, more than 6 months ago/ Sí, hace más de 6 meses (1)  Yes, in the past 6 months/ Sí, en los últimos 6 meses (2) |

# FOLLOW-UP VISITS (MONTHS 6 & 12)

**ANTICIPATED ISS EXPOSURE (Months 6 & 12: ALL top 5 alters)**

**ANTICIPATED ISS EXPOSURE FROM ALTERS**

| **Programming Note:**  For section, list all **TOP 5** alters named for any of the 7 name generator questions. | | |
| --- | --- | --- |
| **The next 3 questions ask about how these people might treat you differently in the FUTURE because of your ethnicity, masculinity, and sexuality.**  **Las siguientes 3 preguntas se refieren a cómo las personas de las que hemos hablado podrían tratarlo de manera diferente en el FUTURO debido a su etnia, masculinidad y sexualidad.** | | |
| VAR name | ITEM | Response Option (value) |
| aANTstigL_M6 | 1. In the **FUTURE**, how likely is it that these people might make negative comments or jokes that bother you, judge you, treat you poorly, or avoid you because you are **Latino**?   En el **FUTURO**, ¿qué probabilidad hay de que estas personas hagan comentarios o chistes negativos sobre los latinos que le molesten, le juzguen, le traten mal o le eviten por ser **latino**? | Very unlikely/ muy improbable (1)  Unlikely/ improbable (2)  it’s possible/ Es posible (3)  Likely/ probable (4)  Very likely/ Muy probable (5) |
| aANTstigM_M6 | 1. In the **FUTURE**, how likely is it that these people might make negative comments or jokes that bother you, judge you, treat you poorly, or avoid you because **you don’t act ‘traditionally’ masculine**?   En el **FUTURO**, ¿qué probabilidad hay de que estas personas hagan comentarios o bromas negativas que le molesten, le juzguen, le traten mal o le eviten porque **no actua de forma “tradicional” masculina**? | Very unlikely/ muy improbable (1)  Unlikely/ improbable (2)  it’s possible/ Es posible (3)  Likely/ probable (4)  Very likely/ Muy probable (5) |
| aANTstigG_M6 | 1. In the **FUTURE**, how likely is it that these people might make negative comments or jokes that bother you, judge you, treat you poorly, or avoid you because you **have sex with other men**?   En el **FUTURO**, ¿qué probabilidad hay de que estas personas hagan comentarios o bromas negativas que le molesten, le juzgen, le traten mal o le eviten porque **tiene relacions sexuales con otros hombres**? | Very unlikely/ muy improbable (1)  Unlikely/ improbable (2)  it’s possible/ Es posible (3)  Likely/ probable (4)  Very likely/ Muy probable (5) |

**ISS AVOIDANCE (Months 6 & 12: all top 5 alters)**

**EGO AVOIDANCE OF ALTERS DUE TO ISS**

| **Programming Note:**  For section **SNI7:** list all **TOP 5** alters named for any of the following name generator questions (i.e., selected for nTalk56 OR nHealth56 OR nSex56 OR nDrug56 OR nHang56 OR nReject56 OR nAvoid56. | | |
| --- | --- | --- |
| **The next 3 questions ask whether YOU have ever wanted to avoid or not interact with any of these people because of their views towards Latinos, masculinity, or sexuality.**  **Las siguientes 3 preguntas se refieren a si USTED alguna vez ha querido evitar o no interactuar con alguna de estas personas debido a sus puntos de vista hacia los latinos, la masculinidad o la sexualidad.** | | |
| VAR name | ITEM | Response Option (value) |
| EgoAvoidL6 | 1. Which of these people have **YOU** wanted to **avoid or not interact** **with** because of their views on **Latinos**?   ¿Con cuál de estas personas ha querido **evitar o no interactuar** debido a sus opiniones sobre **los(as) latinos(as)**? | Never/ Nunca (0)  Yes, more than 6 months ago/ Sí, hace MÁS de 6 meses (1)  Yes, in the past 6 months/ Sí, en los ÚLTIMOS 6 meses (2) |
| EgoAvoidM6 | 1. Which of these people have **YOU** ever wanted to **avoid or not interact** **with** because their views on **how you express your masculinity**?   ¿Con cuál de estas personas ha querido evitar alguna vez o no interactuar debido a sus puntos de vista sobre **cómo expresa su masculinidad**? | Never/ Nunca (0)  Yes, more than 6 months ago/ Sí, hace más de 6 meses (1)  Yes, in the past 6 months/ Sí, en los últimos 6 meses (2) |
| EgoAvoidG6 | 1. Which of these people have **YOU** ever wanted to **avoid or not interact** **with** because of their views on **men who have sex with other men**?   ¿Con cuál de estas personas ha querido evitar o no interactuar debido a las opiniones que tienen sobre **los hombres que tienen sexo con otros hombres**? | Never/ Nunca (0)  Yes, more than 6 months ago/ Sí, hace más de 6 meses (1)  Yes, in the past 6 months/ Sí, en los últimos 6 meses (2) |

**ENACTED ISS EXPOSURE (Months 6 & 12: All top 5 alters)**

**ENACTED ISS EXPOSURE FROM ALTERS**

| **Programming Note:**  For section, list all **TOP 5** alters named for any of the 7 name generator questions. | | |
| --- | --- | --- |
| **Now, we want to ask 3 questions, similar to the ones we asked previously. These questions will ask how often these people have treated you differently in the PAST because of your ethnicity, masculinity, and sexuality.**  **Ahora, queremos hacer 3 preguntas, similares a las que hicimos anteriormente. Estas preguntas se refieren a la frecuencia con la que estas personas de las que hemos hablado le han tratado de manera diferente en el PASADO debido a su etnia, masculinidad y sexualidad.** | | |
| VAR name | ITEM | Response Option (value) |
| aENTstigL_M6 | 1. In the **PAST**, how often has each of these people made negative comments or jokes that bother you, judged you, treated you poorly, or avoided you because you are **Latino?**   En el **PASADO**, ¿con qué frecuencia cada una de estas personas ha hecho comentarios o bromas negativas que le han molestado, le han juzgado, le han tratado mal o le han evitado por ser **latino**? | Never/ Nunca (1)  Not Often/ raramente (2)  Somewhat Often/ algunas veces (3)  Often/ frecuentemente (4)  Very Often/ muy frecuentemente (5) |
| aENTstigM_M6 | 1. In the **PAST**, how often has each of these people made negative comments or jokes that bother you, judged you, treated you poorly, or avoided you because you **don’t act ‘traditionally’ masculine?**   En el **PASADO**, ¿con qué frecuencia cada una de estas personas ha hecho comentarios o bromas negativas que le han molestado, le han juzgado, le han tratado mal o le han evitado porque **no actúa de forma “tradicionalmente” masculina**? | Never/ Nunca (1)  Not Often/ raramente (2)  Somewhat Often/ algunas veces (3)  Often/ frecuentemente (4)  Very Often/ muy frecuentemente (5) |
| aENTstigG_M6 | 1. In the **PAST**, how often has each of these people made negative comments or jokes that bother you, judged you, treated you poorly, or avoided you because you **have sex with other men**?   En el **PASADO**, ¿con qué frecuencia cada una de estas personas ha hecho comentarios o bromas negativas que le han molestado, le han juzgado,, le han tratado mal o le han evitado porque **tiene relaciones sexuales con otros hombres**? | Never/ Nunca (1)  Not Often/ raramente (2)  Somewhat Often/ algunas veces (3)  Often/ frecuentemente (4)  Very Often/ muy frecuentemente (5) |

**INTERSECTIONAL SUPPORT (Months 6 & 12: all top 5 alters)**

**INTERSECTIONAL AFFIRMATION AND SUPPORT FROM ALTERS**

| **Programming Note:**  For section **SNI 9:** list all **TOP 5** alters named for any of the following name generator questions (i.e., selected for nTalk56 OR nHealth56 OR nSex56 OR nDrug56 OR nHang56 OR nReject56 OR nAvoid56. | | |
| --- | --- | --- |
| **The questions from this section ask about positive experiences you have had with these people related to your ethnicity, masculinity, and sexuality.**  **Las preguntas de esta sección se refieren a experiencias positivas que ha tenido con estas personas relacionadas con su origen étnico, masculinidad y sexualidad.** | | |
| VAR name | ITEM | Response Option (value) |
| aAffirmL6 | 1. Which of these people have made you **think or believe** that they support your **Latino** identity? *This could be verbal or non-verbal gestures of support.*   ¿Cuáles de estas personas le han hecho **pensar o creer** que apoyan su identidad **latina**? *Pueden ser gestos de apoyo verbales o no verbales.* | Never/ Nunca (0)  Yes, more than 6 months ago/ Sí, hace MÁS de 6 Meses (1)  Yes, in the past 6 months/ Sí, en los ÚLTIMOS 6 Meses (2) |
| aAffirmM6 | 1. Which of these people have made you **think or believe** that they support how you **express your masculinity**? *This could be verbal or non-verbal gestures of support.*   ¿Cuáles de estas personas le han hecho **pensar o creer** que apoyan la forma en que **expresa su masculinidad**? *Pueden ser gestos de apoyo verbales o no verbales*. | Never/ Nunca (0)  Yes, more than 6 months ago/ Sí, hace MÁS de 6 Meses (1)  Yes, in the past 6 months/ Sí, en los ÚLTIMOS 6 Meses (2) |
| aAffirmG6 | 1. Which of these people have made you **think or believe** that they support you as a **man who has sex with other men**? *This could be verbal or non-verbal gestures of support.*   ¿Cuáles de estas personas le han hecho **pensar o creer** que le apoyan como **hombre que tiene sexo con otros hombres**? *Pueden ser gestos de apoyo verbales o no verbales*. | Never/ Nunca (0)  Yes, more than 6 months ago/ Sí, hace MÁS de 6 Meses (1)  Yes, in the past 6 months/ Sí, en los ÚLTIMOS 6 Meses (2) |
| **People who made you feel welcomed or embraced…**  **Personas que le hicieron sentir bienvenido o aceptado ...** | | |
| aWelcomeL6 | 1. Which of these people have **made you feel welcome or embraced** your experiences as someone who is **Latino**?   ¿Cuál de estas personas le ha **hecho** **sentir bienvenido o aceptado** sus experiencias como **latino**? | Never/ Nunca (0)  Yes, more than 6 months ago/ Sí, hace MÁS de 6 Meses (1)  Yes, in the past 6 months/ Sí, en los ÚLTIMOS 6 Meses (2) |
| aWelcomeM6 | 1. Which of these people have **made you feel welcome or embraced** how you **express your masculinity**?   ¿Cuál de estas personas le ha **hecho sentir bienvenido o aceptado** cómo **expresa su masculinidad**? | Never/ Nunca (0)  Yes, more than 6 months ago/ Sí, hace MÁS de 6 Meses (1)  Yes, in the past 6 months/ Sí, en los últimos 6 meses (2) |
| aWelcomeG6 | 1. Which of these people have **made you feel welcome or embraced** your experiences as a **man who has sex with other men**?   ¿Cuál de estas personas le ha **hecho sentir bienvenido o aceptado** la manera cómo expresa experiencias como **hombre que tiene sexo con otros hombres**? | Never/ Nunca (0)  Yes, more than 6 months ago/ Sí, hace más de 6 meses (1)  Yes, in the past 6 months/ Sí, en los últimos 6 meses (2) |
